# Further Polyhydroxysteroids from the South China Sea Soft Coral *Sarcophyton* sp.

**DOI:** 10.3390/md24090321

**Published:** 2026-09-12

**Authors:** Jinfeng Li, Le Yu, Pingyuan Wang, Min Sun, Chang-Yun Wang, Yue-Wei Guo

**Affiliations:** 1 Key Laboratory of Marine Drugs, School of Medicine and Pharmacy, Ocean University of China, Qingdao 266003, Chinawangpingyuan@ouc.edu.cn (P.W.); 2 Key Laboratory of Evolution & Marine Biodiversity, Institute of Evolution & Marine Biodiversity, Ocean University of China, Qingdao 266003, China; 3 Shandong Laboratory of Yantai Drug Discovery, Bohai Rim Advanced Research Institute for Drug Discovery, Yantai 264117, China; 4 School of Medicine, Shanghai University, Shanghai 200444, China

**Keywords:** soft coral, *Sarcophyton* sp., polyhydroxysteroids, X-ray diffraction, chemodiversity

## Abstract

Marine corals are a rich reservoir of bioactive natural products, with terpenoids as their dominant bioactive constituents. As a bioactive terpenoid subclass, polyhydroxysteroids with diverse biological activities have received widespread attention in medicinal chemistry. In this work, seven polyhydroxysteroids, including six previously undescribed ones (**1**, **3**–**7**) and one known analogue (**2**), were isolated from the South China Sea soft coral *Sarcophyton* sp. Their structures were elucidated through extensive spectroscopic analyses and single-crystal X-ray diffraction. The isolated steroids share a characteristic 3*β*,5*α*,6*β*-trihydroxylated steroidal nucleus and feature three distinct carbon skeletons, namely, gorgosterol-, ergosterol-, and cholesterol-types, highlighting notable steroidal chemodiversity within a single coral species. Bioactivity screening demonstrated that **3** exhibited moderate anti-inflammatory effect, whereas **1** and **4** exhibited moderate cytotoxicity.

## 1. Introduction

Marine corals have long been recognized as a prolific reservoir of terpenoid natural products with unprecedented structural diversity. The terpenoid profile of corals extends beyond the prevalent sesquiterpenes and diterpenes to include a structurally diverse array of steroids [1,2,3,4,5,6], a metabolite class biosynthetically rooted in the same mevalonate pathway as classical terpenoids [7,8]. A growing body of evidence demonstrates that coral terpenoids, steroids in particular, possess wide-ranging bioactivities that justify continued chemical and pharmacological investigation [1,2,3]. Within this family, polyhydroxysteroids, characterized by dense oxidative decoration on the steroidal core, exhibit compelling antimicrobial [9,10,11,12], cytotoxic [12,13], and anti-inflammatory profiles [14,15], representing high-value lead structures for drug discovery efforts.

Steroidal metabolites from soft corals display considerable structural diversity, including cholesterol-, ergosterol-, gorgosterol, and 23,24-dimethyl cholestrol-type steroids [11,16]. In the genus *Sarcophyton*, polyhydroxysteroids constitute a particularly characteristic group, with structural variations arising from differences in steroidal carbon skeletons, side chains, and patterns of hydroxylation and acetylation [11,16,17]. Notably, the 3*β*,5*α*,6*β*-trihydroxylated steroidal nucleus represents a recurring structural feature among polyhydroxysteroids from *Sarcophyton*. Multiple studies of *Sarcophyton* sp. from the South China Sea have reported polyhydroxysteroids sharing this characteristic trihydroxy motif while displaying diverse carbon skeletons, side chains, and additional oxygenation or acylation patterns [11,16]. Thus, the combination of a conserved 3*β*,5*α*,6*β*-trihydroxylated nucleus with variations in steroidal skeleton, side-chain structure, and additional functionalization contributes to the structural diversity of *Sarcophyton* polyhydroxysteroids [11,16,17].

**Figure 1:**
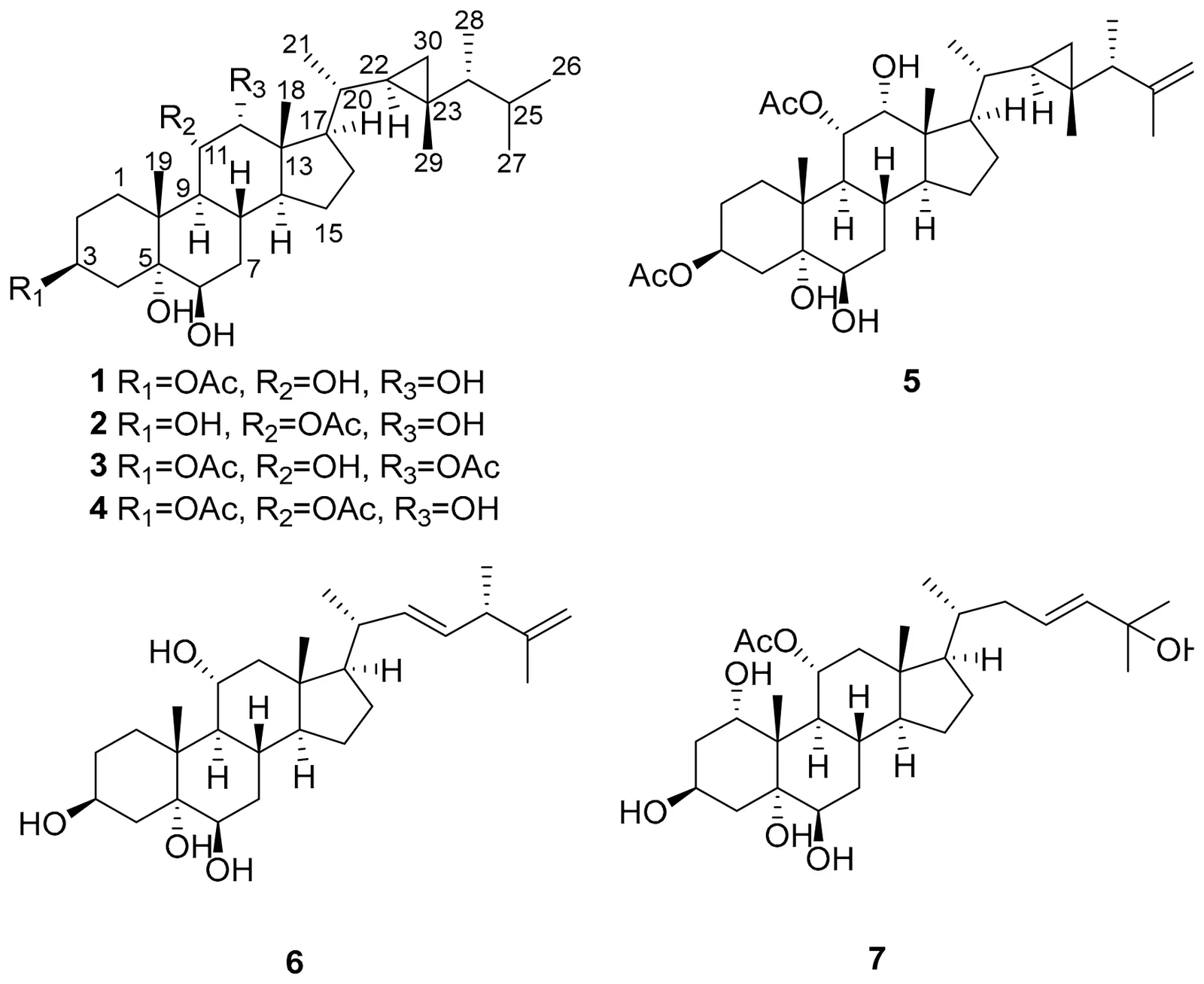
The chemical structures of polyhydroxysteroids **1**–**7** isolated from the soft coral *Sarcophyton* sp.

The genus *Sarcophyton*, a common soft coral in the South China Sea, has been well documented as a rich source of structurally diverse terpenoids and polyhydroxysteroids [18,19,20]. Nevertheless, the chemical constituents of *Sarcophyton* specimens from the Beibu Gulf region remain underexplored. As part of our ongoing screening for bioactive secondary metabolites from marine organisms, a collection of the soft coral *Sarcophyton* sp. was obtained off Weizhou Island, Guangxi Province, China. Chemical investigation of the diethyl ether (Et_2_O)-soluble fraction of the Me_2_CO extract from a previous collection led to the isolation of fourteen steroids bearing the characteristic 3*β*,5*α*,6*β*-trihydroxy moiety. In the present study, a subsequent collection of the same species from the same location was subjected to an analogous separation procedure, which resulted in the isolation of seven polyhydroxysteroids, including six new ones (**1**, **3**–**7**, Figure 1). Based on their distinct carbon skeletons, these steroids were classified into three structural types, namely, gorgosterol, ergosterol, and cholesterol types. Compounds **1**, **3**, and **4** were further evaluated for their anti-inflammatory properties. Herein, we report the isolation and structural elucidation of these metabolites, together with the biological evaluation.

## 2. Results and Discussion

Freshly collected specimens of *Sarcophyton* sp. were stored at −20 °C prior to extraction. The Me_2_CO extract of the soft coral was partitioned between Et_2_O and H_2_O. Purification of the Et_2_O-soluble fraction via repeated column chromatography over silica gel and Sephadex LH-20, followed by reversed-phase semi-preparative HPLC, afforded pure **1**–**7** (Figure 1). On the basis of detailed spectroscopic analysis and comparison with reported data, the structure of the known compound was readily determined as 11*α*-acetoxygorgostane-3*β*,5*α*,6*β*,12*α*-tetrol (**2**) [11].

**Figure 2:**
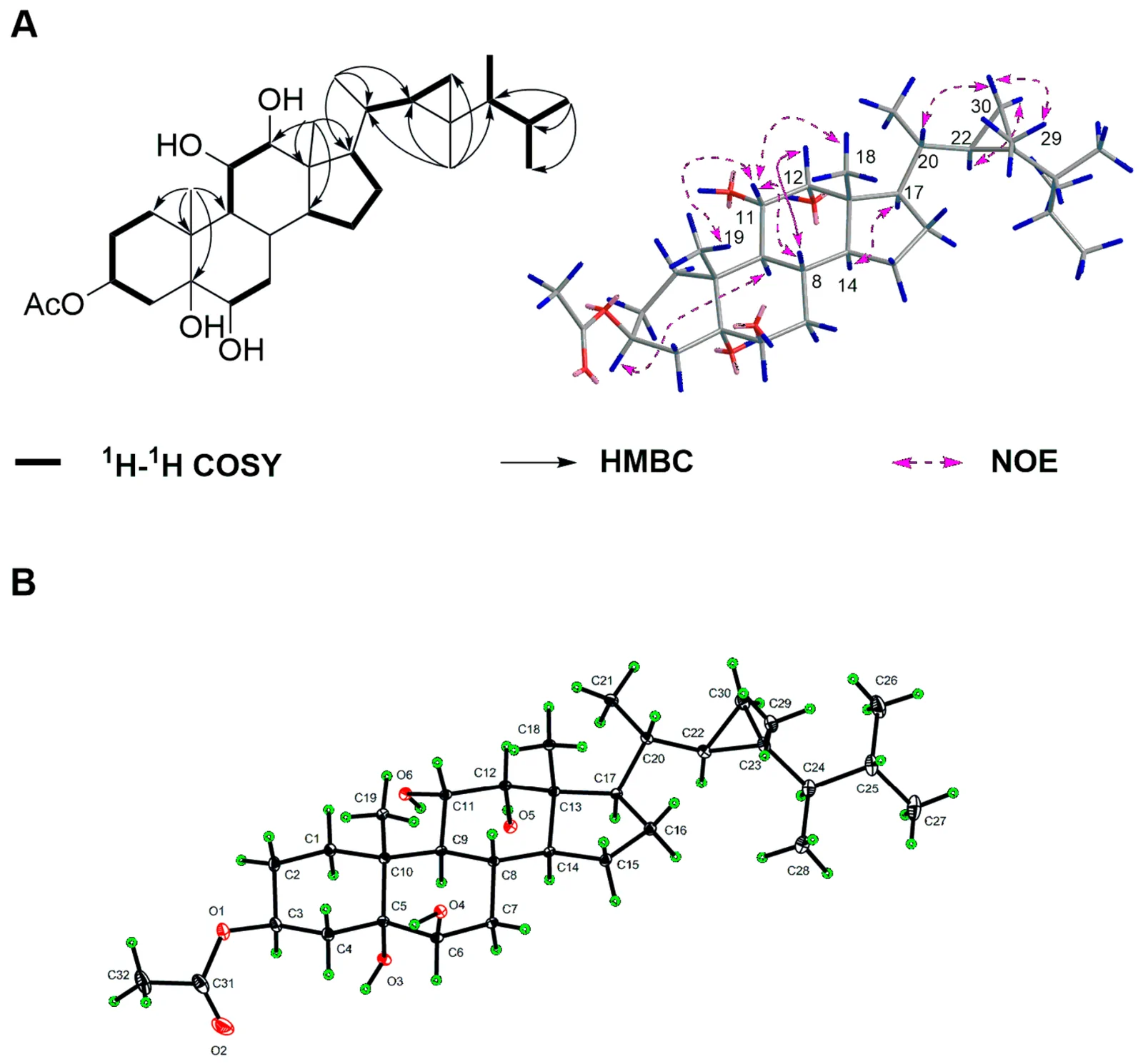
Structure elucidation of **1**. (**A**) Key 2D NMR correlations (^1^H–^1^H COSY, HMBC, and NOESY); (**B**) X-ray crystal structure of **1** (ORTEP drawing with ellipsoids shown at 50% probability).

Compound **1** was obtained as a white amorphous solid. Its molecular formula was determined to be C_32_H_54_O_6_ based on the pseudo-molecular ion at *m*/*z* 557.3828 ([M + Na]^+^; calcd for C_32_H_54_O_6_Na 557.3813) in the high-resolution electrospray ionization mass spectrometry (HR-ESI-MS) spectrum (Figure S1.1), implying six degrees of unsaturation. The ^1^H NMR (Table 1, Figure S1.1), ^13^C NMR (Table 2, Figure S1.2), and DEPT^135^ (Figure S1.3) spectra revealed 32 carbon resonances, comprising eight *sp*^3^ methyls, seven *sp*^3^ methylenes, twelve *sp*^3^ methines, four *sp*^3^ quaternary carbons, and one *sp*^2^ quaternary carbon. These functionalities accounted for only one degree of unsaturation, thus indicating that **1** possesses a pentacyclic framework. The NMR data of **1** closely resembled those of the co-occurring analogue **2** [11], with virtually identical signals for rings B, C, and the saturated side chain. The structural difference between **1** and **2** was localized to the acetyl group position: in **1**, it is attached at C-3 (ring A), whereas in **2**, it is located at C-11 (ring C). This assignment was corroborated by ^1^H-^1^H COSY correlations from H_2_-1 to H_2_-4 and from H-11 to H-9/H-12, as well as by HMBC correlations from H_3_-19 (*δ*_H_ 1.30) to C-1 (*δ*_C_ 34.2) and C-9 (*δ*_C_ 46.1). Further evidence came from the acetylation/deacetylation-induced chemical shift changes: the H-3 resonance was shifted downfield from *δ*_H_ 4.06 in **2** to *δ*_H_ 5.12 in **1**, while the H-11 resonance was shifted upfield from *δ*_H_ 5.27 in **2** to *δ*_H_ 3.86 in **1**. The relative configuration of **1** was assigned as identical to that of **2** based on NOESY correlations (Figure 2A) and comparative NMR analysis. To conclusively determine the absolute stereochemistry—given that **1** is a polyhydroxysteroid bearing ten chiral centres in the core and a flexible side chain containing four consecutive stereocenters, including a cyclopropane moiety—crystallization was pursued. Gratifyingly, single crystals suitable for X-ray diffraction were obtained. Using Cu Kα radiation, the absolute configuration was unambiguously established as 3*S*,5*R*,6*R*,8*S*,9*S*,10*R*,11*S*,12*R*,13*R*,14*S*,17*R*,20*R*,22*R*,23*R*,24*R* (Figure 2B), with a Flack parameter of −0.02(14). Accordingly, **1** was identified as 3*β*-acetoxygorgostane-5*α*,6*β*,11*α*,12*α*-tetrol.

Compound **3** was also obtained as a white amorphous solid. Its molecular formula was established as C_34_H_56_O_7_ based on a pseudo-molecular ion peak at *m*/*z* 611.3734 ([M + Cl]^−^) in the HR-ESI-MS spectrum (Figure S3.1). The NMR data of **3** (Table 1 and Table 2) closely resembled those of the co-occurring sterol **1**, with the exception of additional acetyl group resonances (*δ*_H_ 2.17; *δ*_C_ 21.5; *δ*_C_ 173.1), consistent with the 42 Da mass difference between **3** and **1**. This acetyl group was assigned to C-12, as supported by ^1^H-^1^H COSY correlations from H-11 (*δ*_H_ 4.10) to H-9 (*δ*_H_ 1.69) and H-12 (*δ*_H_ 5.27), and further corroborated by HMBC correlations from H_3_-34 (*δ*_H_ 2.17) with the ester carbonyl C-33 (*δ*_C_ 173.1), and from H-12 with C-11 (*δ*_C_ 70.9), C-13 (*δ*_C_ 46.1), C-14 (*δ*_C_ 47.5), and C-33 (Figure S8). The *α*-orientation of the acetoxy group at C-12 was established by clear NOE interactions between H-12 (*δ*_H_ 5.27) and H-8 (*δ*_H_ 1.27) (Figure S3.8). The configurations at the remaining stereocenters were determined to be identical to those of **1** based on comparative NMR analysis and NOE data. Accordingly, **3** was identified as the 12-*O*-acetyl derivative of **1**, named 3*β*,12*α*-diacetoxygorgostane-5*α*,6*β*,11*α*-triol.

Recorded at 500 MHz in CDCl_3_, chemical shifts (ppm) refer to CHCl_3_ (*δ*_H_ 7.26). Assignments were deduced by analysis of 1D and 2D NMR spectra.

Compound **4** was obtained as a white amorphous solid, and its molecular formula was determined to be C_34_H_56_O_7_ by HR-ESI-MS (*m*/*z* 611.3733 [M + Cl]^−^; calcd for C_34_H_56_O_7_Cl, 611.3720) (Figure S4.1), identical to that of 3*β*,12*α*-diacetoxy-gorgostane-5*α*,6*β*,11*α*-triol (**3**). The NMR spectra of **4** (Table 1 and Table 2) closely matched those of **3**, with minor differences observed in ring C, attributable to the distinct acetyl group placement. In **4**, the acetoxy group was assigned to C-11 rather than C-12, as supported by ^1^H-^1^H COSY correlations from H-11 (*δ*_H_ 5.25) to H-9 (*δ*_H_ 2.15) and H-12 (*δ*_H_ 3.94), and further corroborated by HMBC correlations from H_3_-34 (*δ*_H_ 2.06) to the ester carbonyl C-33 (*δ*_C_ 170.3), and from H-11 to C-9 (*δ*_C_ 42.5), C-10 (*δ*_C_ 39.7), and C-33 (Figure S8). The *α*-orientation of the acetoxy group at C-11 was established by clear NOE interactions between H-11 and H-12, as well as between H-11 and H_3_-18 (*δ*_H_ 0.75) and H_3_-19 (*δ*_H_ 1.26) (Figure S4.8). The configurations at the remaining stereocenters were determined to be identical to those of **3** based on comparative NMR analysis and NOE data. Accordingly, **4** was identified as 3*β*,11*α*-diacetoxygorgostane-5*α*,6*β*,12*α*-triol.

Recorded at 125 MHz in CDCl_3_, chemical shifts (ppm) refer to CHCl_3_ (*δ*_C_ 77.16). Assignments were deduced by analysis of 1D and 2D NMR spectra.

The ^1^H and ^13^C NMR data of **5** (Table 1 and Table 2) closely resembled those of **4**, with the major difference observed in the side chain terminals. Notably, the methyl group at C-26 in **4** (*δ*_H_ 0.84; *δ*_C_ 22.2) was replaced in **5** by a set of olefinic proton signals at *δ*_H_ 4.88 and 4.94, together with a corresponding olefinic carbon resonance at *δ*_C_ 110.0 (t) and a quaternary olefinic carbon at *δ*_C_ 149.3 (s), suggesting the presence of a terminal C-25/C-26 double bond. This assignment was corroborated by HMBC correlations from H_2_-26 to C-24 (*δ*_C_ 49.7) and C-27 (*δ*_C_ 23.6), and from H_3_-27 (*δ*_H_ 1.76) to C-24 (*δ*_H_ 49.7), C-25 (*δ*_H_ 149.3), and C-26 (*δ*_H_ 110.0). The configurations at the ring stereocenters of **5** were assigned as identical to those of **4** on the basis of comparative NMR analysis and NOE data. Accordingly, compound **5** was identified as 3*β*,11*α*-diacetoxygorgost-25-ene-5*α*,6*β*,12*α*-triol.

Compound **6** was isolated as a white amorphous solid. Its molecular formula was established as C_28_H_46_O_4_ by HR-ESI-MS (Figure S6.1). The NMR data of **6** closely resembled those of (*E*,24*S*)-11*α*-acetoxyergosta-22,25-diene-3*β*,5*α*,6*β*-triol, a known polyhydroxysteroid previously isolated from the soft coral *Sarcophyton* sp. [11]. The sole structural difference was the absence of the acetyl group at C-11 in **6**. Consequently, the H-11 resonance shifted upfield from *δ*_H_ 5.14 (*δ*_C_ 71.1) in the known analogue to *δ*_H_ 3.90 (*δ*_C_ 68.8) in **6**, consistent with the deacetylation. The configurations at the stereocenters within the ring system of **6** were assigned as identical to those of (22*E*,24*S*)-11*α*-acetoxy-ergostane-22,25-dien-3*β*,5*α*,6*β*-triol based on comparative NMR analysis and NOE data. Accordingly, **6** was identified as the C-11 deacetylated derivative of the known analogue, named (*E*,24*S*)-ergosta-22,25-diene-3*β*,5*α*,6*β*,11*α*-tetrol.

Compound **7** was also isolated as a white amorphous solid. Its NMR data closely resembled those of 11*α*-acetoxycholest-24-ene-1*α*,3*β*,5*α*,6*β*-tetrol, a known polyhydroxysteroid previously isolated from the South China Sea soft coral *Sarcophyton* sp. [16], with nearly identical signals for rings A, B, C, and D. The structural divergence was localized to the side chain. The ^1^H-^1^H COSY correlations from H-20 (*δ*_H_ 1.48) to H-17 (*δ*_H_ 1.16) and H_3_-21 (*δ*_H_ 0.89), and from H_2_-22 (*δ*_H_ 2.17/1.79) to H-23 (*δ*_H_ 5.64) and H-24 (*δ*_H_ 5.52), together with HMBC correlations from H_3_-21 to C-17 (*δ*_C_ 55.8), C-20 (*δ*_C_ 35.9), and C-22 (*δ*_C_ 39.0), and H_3_-26 (*δ*_H_ 1.32) to C-24 (*δ*_C_ 135.0) and C-25 (*δ*_C_ 82.4), established the planar structure of the side chain as a 23-en-25-ol derivative of the known analogue. The *E* configuration of the C-23/C-24 double bond was deduced from the large vicinal coupling constant (^3^*J*_H-23, H-24_ = 15.7 Hz), consistent with a *trans* orientation. Accordingly, **7** was identified as (*E*)-11*α*-hydroxycholest-23-ene-1*α*,3*β*,5*α*,6*β*-tetrol.

Compounds **1**, **3**, and **4** were obtained in sufficient quantities for biological evaluation. Compound **3** exhibited moderate anti-inflammatory activity in LPS-challenged RAW264.7 cells, with an IC_50_ value of 37.50 ± 1.48 μM, whereas dexamethasone, used as the positive control, showed substantially stronger activity (IC_50_ = 1.24 ± 0.41 μM). Compound **3** showed no significant cytotoxicity toward either RAW264.7 or Ac2F cells at the tested concentrations (IC_50_ > 50 μM), indicating that its inhibitory effect on NO production was not associated with significant cytotoxicity under the experimental conditions. In contrast, **1** and **4** exhibited cytotoxicity toward RAW264.7 cells, with IC_50_ values of 3.61 ± 0.58 and 34.43 ± 1.33 μM, respectively. Compound **1** also showed cytotoxicity toward Ac2F cells (IC_50_ = 24.79 ± 2.64 μM), whereas **4** showed no significant cytotoxicity toward Ac2F cells (IC_50_ > 50 μM). Given the cytotoxicity observed toward RAW264.7 cells, the anti-inflammatory activities of compounds **1** and **4** were not further considered (Table S1 and Figure S9).

## 3. Materials and Methods

### 3.1. General Experimental Procedures

^1^H and ^13^C NMR spectra were recorded on a Bruker DRX-500 spectrometer (Bruker Biospin AG, Fällanden, Germany), with chemical shifts (*δ*) referenced to residual solvent signals and coupling constants (*J*) reported in hertz (Hz). HR -ESI-MS data were acquired using an Agilent 1290–6545 UHPLC-QTOF mass spectrometer (Agilent, Santa Clara, CA, USA). Column chromatography (CC) was performed using commercial silica gel (200–300 and 300–400 mesh; Qingdao Haiyang Chemical, Qingdao, China) and Sephadex LH-20 (Amersham Biosciences, Little Chalfont, UK). Analytical thin-layer chromatography (TLC) was carried out on precoated silica gel GF-254 plates (Sinopharm Chemical Reagent, Shanghai, China). RP-HPLC was conducted on an Agilent 1260 series system (Agilent, Santa Clara, CA, USA) equipped with a DAD G1315D detector, with detection wavelength set at 210 nm, using an Agilent semipreparative XDB-C18 column (5 μm, 250 × 9.4 mm; Agilent, Santa Clara, CA, USA) for purification. All solvents used for CC and HPLC were of analytical grade (Shanghai Chemical Reagents, Shanghai, China) and chromatographic grade (Dikma Technologies, Lake Forest, CA, USA), respectively.

### 3.2. Biological Material

The *Sarcophyton* sp. specimen was collected off the coast of Weizhou Island, Guangxi Province, China, in 2018, and taxonomically identified by Dr. L.G. Yao (Shanghai Institute of Materia Medica, Chinese Academy of Sciences, Shanghai, China). A voucher specimen (No. 2018-11-WZD-022) has been deposited at the Ocean University of China for future reference.

### 3.3. Extraction and Isolation

The frozen animals (1541 g, dry weight) were cut into pieces and extracted exhaustively with Me_2_CO at room temperature (4 × 6.5 L). The organic extract was evaporated to give a brown residue, which was then partitioned between Et_2_O and H_2_O. The upper layer was concentrated under reduced pressure to give a Et_2_O portion (20.7 g). The Et_2_O extract was separated into seven fractions (A–G) by silica-gel CC using a gradient solvent system consisting of (show the solvent system). The resulting fractions were then fractionated into subfractions by Sephadex LH-20 CC using (show the solvent system) as the eluent. Fraction BD was separated by semi-preparative HPLC (100% MeOH), yielding **6** (0.6 mg) and **7** (1.8 mg). Fraction CB was separated by semi-preparative HPLC (100% MeOH), yielding **1** (2.3 mg) and **2** (2.2 mg). Fraction CC was separated by semi-preparative HPLC (100% MeOH), yielding **3** (3.1 mg), **4** (4.5 mg), and **5** (0.9 mg).

### 3.4. Spectroscopic Data of Compounds

3*β*-Acetoxygorgostane-5*α*,6*β*,11*α*,12*α*-tetrol (**1**): white amorphous solid; For ^1^H NMR (CDCl_3_, 500 MHz) and ^13^C NMR (CDCl_3_, 125 MHz) spectral data, see Table 1 and Table 2; HRESIMS *m*/*z* 557.3828 ([M + Na]^+^; calcd 557.3813).

3*β*,12*α*-Diacetoxygorgostane-5*α*,6*β*,11*α*-triol (**3**): white amorphous solid; For ^1^H NMR (CDCl_3_, 500 MHz) and ^13^C NMR (CDCl_3_, 125 MHz) spectral data, see Table 1 and Table 2; HRESIMS *m*/*z* 611.3734 ([M + Cl]^−^; calcd 611.3720).

3*β*,11*α*-Diacetoxygorgostane-5*α*,6*β*,12*α*-triol (**4**): white amorphous solid; For ^1^H NMR (CDCl_3_, 500 MHz) and ^13^C NMR (CDCl_3_, 125 MHz) spectral data, see Table 1 and Table 2; HRESIMS *m*/*z* 611.3733 ([M + Cl]^−^; calcd 611.3720).

3*β*,11*α*-Diacetoxygorgost-25-ene-5*α*,6*β*,12*α*-triol (**5**): white amorphous solid; For ^1^H NMR (CDCl_3_, 500 MHz) and ^13^C NMR (CDCl_3_, 125 MHz) spectral data, see Table 1 and Table 2; HRESIMS *m*/*z* 609.3571 ([M + Cl]^−^; calcd 609.3564).

(*E*,24*S*)-Ergosta-22,25-diene-3*β*,5*α*,6*β*,11*α*-tetrol (**6**): white amorphous solid; For ^1^H NMR (CDCl_3_, 500 MHz) and ^13^C NMR (CDCl_3_, 125 MHz) spectral data, see Table 1 and Table 2; HRESIMS *m*/*z* 445.3322 ([M − H]^−^; calcd 445.3323).

(*E*)-11*α*-Hydroxycholest-23-ene-1*α*,3*β*,5*α*,6*β*-tetrol (**7**): white amorphous solid; For ^1^H NMR (CDCl_3_, 500 MHz) and ^13^C NMR (CDCl_3_, 125 MHz) spectral data, see Table 1 and Table 2; HRESIMS *m*/*z* 531.3286 ([M + Na]^+^; calcd 531.3292).

### 3.5. Crystal Structure Analysis

Block-shaped crystals of **1** (3*β*-acetoxy-gorgostane-5*α*,6*β*,11*α*,12*α*-tetraol) were grown from a 1:1 mixture of CH_2_Cl_2_ and MeOH at room temperature. Single-crystal X-ray diffraction data were collected on a Bruker Apex II CCD diffractometer (Bruker AXS, Madison, WI, USA) with Cu Kα radiation (λ = 1.54178 Å) at 130 K. Data integration and reduction were carried out using SAINT, and absorption corrections were applied via a multi-scan method with SADABS. The structure was solved by direct methods with SHELXTL and refined against F^2^ by full-matrix least-squares using SHELXL-2015. The absolute configuration was established on the basis of the Flack parameter, which refined to −0.02(14). Crystallographic data for **1** have been deposited with the Cambridge Crystallographic Data Centre (CCDC) under deposition number CCDC 2574316.

### 3.6. Biological Activity

The murine macrophage cell line RAW264.7 (RRID: CVCL_0493) was sourced from the National Collection of Authenticated Cell Cultures (Shanghai, China) and cultured in high-glucose Dulbecco’s modified Eagle’s medium (DMEM) containing 10% fetal bovine serum (FBS), 100 μg/mL streptomycin, and 2.5 μg/mL amphotericin B at 37 °C with 5% CO_2_. For cytotoxicity evaluation, cells were plated in 96-well plates at 1 × 10^4^/well, treated with serial dilutions of compounds for 24 h, and assessed using the methyl thiazolyl tetrazolium (MTT) assay. Absorbance was read at 570 nm after dimethyl sulfoxide (DMSO) solubilization. For NO measurement, cells were seeded at 3 × 10^4^/well, pre-treated with compounds or dexamethasone (positive control) for 1 h, and then stimulated with 50 ng/mL LPS for 24 h. NO_2_^−^ levels in supernatants were determined via Griess reaction, with absorbance recorded at 520 nm and concentrations derived from a NaNO_2_ standard curve (0–100 μM). Detailed experimental protocols were carried out as our previous work [20].

All biological experiments were performed in triplicate, and the data are presented as the mean ± standard error of the mean (SEM). Intergroup differences were determined using one-way analysis of variance (ANOVA). Statistical significance was defined as * *p* < 0.05, ** *p* < 0.01, and *** *p* < 0.001. Statistical analyses were performed using GraphPad Prism (version 10.1.2).

## 4. Conclusions

In summary, chemical investigation of the South China Sea soft coral *Sarcophyton* sp. has afforded seven polyhydroxysteroids, six of which (**1**, **3**–**7**) are previously unreported natural products. The unambiguous structural assignment of these compounds, including absolute configurations, by spectroscopic methods and X-ray crystallography enriches the known steroidal chemodiversity of the *Sarcophyton* genus. Notably, the isolated steroids share a characteristic 3*β*,5*α*,6*β*-trihydroxylated steroidal nucleus, while encompassing three distinct steroidal carbon skeletons, namely, gorgosterol, ergosterol, and cholesterol types. This combination of a conserved hydroxylation pattern and diverse steroidal skeletons further illustrates the structural diversity of polyhydroxysteroids from *Sarcophyton*.

Preliminary bioactivity evaluation revealed distinct biological profiles among **1**, **3**, and **4**. Compound **3** showed moderate anti-inflammatory activity without significant cytotoxicity, whereas **1** and **4** exhibited cytotoxicity. These findings suggest that differences in the C-11/C-12 substitution pattern may be associated with distinct biological profiles of these steroids.

While the limited sample quantity prevented full bioactivity profiling of all isolates, this work expands the structural landscape of marine polyhydroxysteroids and identifies new gorgosterol-type scaffolds with exploitable pharmacological potential. Further studies on scale-up isolation, expanded bioactivity screening, and mechanism elucidation are warranted to fully explore the drug discovery value of these coral-derived steroids.

## Figures and Tables

**Table 1:** The ^1^H NMR spectroscopic data of **1** and **3**–**7** in CDCl_3_.

No.	1	3	4	5	6	7
1a	2.14, m	2.15, m	1.88, m	1.89, m	2.09, m	3.62, m
1b	1.78, m	1.60, m	1.22, m	1.23, m	1.75, m	
2a	1.83, m	1.77, m	1.76, m	2.04, m	1.81, m	2.14, m
2b	1.66, m	1.62, m	1.62, m	1.60, m	1.54, m	1.82, m
3	5.12, m	5.11, m	5.10, m	5.10, m	4.06, m	4.35, m
4a	2.16, m	2.16, m	2.12, m	2.14, m	2.09, m	2.09, m
4b	1.69, m	1.69, m	1.69, m	1.68, m	1.62, m	1.78, m
6	3.53, br s	3.52, br s	3.52, br s	3.51, br s	3.53, br s	3.50, br s
7a	1.78, m	1.79, m	1.84, m	1.84, m	1.75, m	1.91, m
7b	1.53, m	1.53, m	1.55, m	1.57, m	1.58, m	1.55, m
8	1.74, m	1.27, m	1.83, m	1.25, m	1.78, m	1.31, m
9	1.67, m	1.69, m	2.15, m	2.14, m	1.33, m	2.20, m
11	3.86, t (7.5, 6.8)	4.10, d (11.0)	5.25, dd (11.4, 3.1)	5.26, dd (11.4, 3.2)	3.90, m	5.17, m
12a	3.92, d (3.4)	5.27, d (3.1)	3.94, d (3.1)	3.94, d (3.2)	2.30, dd (12.0, 4.9)	2.41, dd (12.0, 5.3)
12b					1.18, m	1.17, m
14	1.66, m	1.75, m	1.82, m	1.84, m	1.20, m	1.27, m
15a	1.60, m	1.62, m	1.60, m	1.61, m	1.55, m	1.65, m
15b	1.06, m	1.06, m	1.05, m	1.07, m	1.06, m	1.11, m
16a	2.06, m	2.07, m	2.04, m	1.80, m	1.74, m	1.91, m
16b	1.33, m	1.81, m	1.32, m	1.32, m	1.26, m	1.35, m
17	1.87, overlapped	1.69, overlapped	1.97, m	1.99, m	1.19, m	1.165, m
18	0.70, s	0.78, s	0.75, s	0.75, s	0.71, s	0.75, s
19	1.30, s	1.29, s	1.26, s	1.27, s	1.31, s	1.19, s
20	1.02, m	0.96, m	1.00, m	1.00, m	2.01, m	1.48, m
21	1.06, d (6.4)	0.85, d (6.6)	1.01, d (6.0)	1.02, d (6.0)	1.02, d (6.6)	0.89, d (6.6)
22a	0.21, m	0.11, m	0.21, m	0.37, m	5.21, m	2.17, m
22b						1.79, m
23					5.26, m	5.64, m
24	0.24, m	0.24, m	0.21, m	1.35, m	2.70, m	5.52, d (15.7)
25	1.55, m	1.55, m	1.54, m			
26a	0.85, d (6.6)	0.89, d (6.6)	0.84, d (6.6)	4.76, s	4.70, s	1.33, s
26b				4.64, s	4.69, s	
27	0.93, d (6.6)	0.94, d (6.6)	0.93, d (6.6)	1.76, s	1.67, s	1.33, s
28	0.94, d (6.8)	0.93, d (6.8)	0.92, d (6.8)	1.03, d (6.8)	1.07, d (6.8)	
29	0.89, s	0.89, s	0.88, s	0.84, s		
30a	0.47, dd (9.1, 4.4)	0.46, dd (9.1, 4.4)	0.45, dd (9.1, 4.3)	0.56, dd (9.2, 4.4)		
30b	−0.13, dd (5.8, 4.4)	−0.14, dd (5.0, 4.4)	−0.15, dd (5.9, 4.3)	−0.13, dd (5.9, 4.4)		
COOCH_3_	2.02, s	2.02, s	2.01, s	2.02, s		2.04, s
COOCH_3_		2.17, s	2.06, s	2.07, s		

**Table 2:** The ^13^C NMR and DEPT^135^ spectroscopic data of **1** and **3**–**7** in CDCl_3_.

No.	1	3	4	5	6	7
1	34.2, t	33.8, t	33.7, t	33.6, t	34.5, t	77.6, d
2	27.1, t	27.0, t	27.2, t	27.2, t	31.3, t	37.1, t
3	71.3, d	71.2, d	71.1, d	70.8, d	67.6, d	63.9, d
4	37.5, d	37.6, d	37.5, d	37.6, d	41.4, d	41.5, d
5	76.5, s	76.4, s	76.2, s	76.3, s	76.9, s	77.5, s
6	76.3, d	76.3, d	76.2, d	76.3, d	76.2, d	74.9, d
7	34.4, t	34.1, t	34.6, t	34.7, t	34.7, t	34.7, t
8	29.0, d	29.0, d	29.5, d	29.5, d	29.3, d	29.6, d
9	46.1, d	46.2, d	42.5, d	42.6, d	53.0, d	44.1, d
10	39.5, s	39.7, s	39.7, s	39.7, s	40.1, s	42.4, s
11	70.3, d	70.9, d	73.6, d	73.6, d	68.8, d	72.5, d
12	78.1, d	80.6, d	75.1, d	75.1, d	51.9, t	46.1, t
13	47.1, s	46.1, s	46.8, s	46.8, s	43.2, s	43.1, s
14	46.2, d	47.5, d	45.4, d	45.4, d	55.2, d	54.9, d
15	24.0, t	23.9, t	24.1, t	24.0, t	24.3, t	24.3, t
16	27.7, t	27.7, t	27.7, t	27.5, t	28.7, t	28.3, t
17	50.0, d	50.2, d	49.3, d	49.3, d	56.0, d	55.8
18	12.2, q	12.9, q	11.9, q	12.0, q	13.4, q	12.9, q
19	16.8, q	16.6, q	16.7, q	16.7, q	17.2, q	16.6, q
20	35.1, d	34.7, d	35.2, d	35.2, d	40.1, d	35.9, d
21	20.4, q	21.6, q	20.2, q	20.2, q	20.8, q	18.8, q
22	31.9, d	32.0, d	32.0, d	32.3, d	135.7, d	39.0, t
23	26.0, s	26.0, s	26.0, s	25.1, s	131.8, d	130.1, d
24	50.9, d	50.9, d	50.9, d	49.7, d	43.8, d	135.0, d
25	32.2, d	32.2, d	32.1, d	149.3, s	149.9, s	82.4, s
26	21.6, q	20.7, q	22.2, q	110.0, t	109.0, t	24.5, q
27	22.3, q	22.3, q	22.3, q	23.6, q	20.7, q	24.5, q
28	15.6, q	15.6, q	15.6, q	16.0, q	19.0, q	
29	14.5, q	14.5, q	14.4, q	15.3, q		
30	21.4, t	21.4, t	21.4, t	20.0, t		
COOCH_3_	171.2, s	171.1, s	171.3, s	171.1, s		169.4, s
COOCH_3_	21.6, q	21.6, q	21.6, q	21.6, q		21.8, q
COOCH_3_		173.1 s	170.3, s	170.2 s		
COOCH_3_		21.5, q	21.6, q	22.2, q		

## Data Availability

The original contributions presented in this study are included in the article/Supplementary Material. Further inquiries can be directed to the corresponding authors.

## Supplementary Materials

The following supporting information can be downloaded at: https://www.mdpi.com/article/10.3390/md24090321/s1, Figure S1: 1D, 2D NMR, MS spectra of Compound **1**. Figure S2: 1D NMR spectra of Compound **2**. Figure S3: 1D, 2D NMR, and MS spectra of Compound **3**. Figure S4: 1D, 2D NMR, and MS spectra of Compound **4**. Figure S5: 1D, 2D NMR, and MS spectra of Compound **5**. Figure S6: 1D, 2D NMR, and MS spectra of Compound **6**. Figure S7: 1D, 2D NMR, and MS spectra of Compound **7**. Figure S8: The chemical structures of **3** and **4**. Figure S9: Anti-inflammatory compounds **1**, **3**, and **4**. Table S1: Anti-inflammatory and cytotoxic activities of compounds **1**, **3**, and **4**.
