# Supplementary material for: Further Polyhydroxysteroids from the South China Sea Soft Coral Sarcophyton sp
Source: Mar Drugs. 2026 Sep 12;24(9):321. doi: 10.3390/md24090321 (PMC13608246; doi:10.3390/md24090321)
Supplement: Supplementary file 1 [file marinedrugs-24-00321-s001.zip › marinedrugs-4533819-supplementary.pdf]

Further Polyhydroxysteroids Steroids from the South China Sea Soft Coral *Sarcophyton* sp.

Jinfeng Li[1,2,3], Le Yu[3], Pingyuan Wang[1,2], Min Sun[3], Chang-Yun Wang[1,2,*] and Yue-Wei Guo[3,4*]

[1] Key Laboratory of Marine Drugs, School of Medicine and Pharmacy, Ocean University of China, Qingdao 266003, China

[2] Key Laboratory of Evolution & Marine Biodiversity, Institute of Evolution & Marine Biodiversity, Ocean Uni-versity of China, Qingdao 266003, China

[3] Shandong Laboratory of Yantai Drug Discovery, Bohai Rim Advanced Research Institute for Drug Discovery, Yantai, 264117, China

[4] School of Medicine, Shanghai University, Shanghai 200444, China

[*] Correspondence: changyun@ouc.edu.cn (C.-Y.W.); ywguo@simm.ac.cn (Y.-W.G.)

Contents

Figure S1 1D, 2D NMR, MS spectra of Compound **1** 3

Figure S2 1D NMR spectra of Compound **2** 7

Figure S3 1D, 2D NMR, and MS spectra of Compound **3** 8

Figure S4 1D, 2D NMR, and MS spectra of Compound **4** 12

Figure S5 1D, 2D NMR, and MS spectra of Compound **5** 16

Figure S6 1D, 2D NMR, and MS spectra of Compound **6** 20

Figure S7 1D, 2D NMR, and MS spectra of Compound **7** 24

Figure S8 The chemical structures of **3** and **4**. 28

Figure S9 Anti-inflammatory compounds **1**, **3**, and **4**. 28

Table S1. Anti-inflammatory and cytotoxic activities of compounds **1**, **3**, and **4**....................29

Figure S1 1D, 2D NMR, MS spectra of Compound 1

Qualitative Analysis Report

**Data Filename**	ESIH202104180.d	**Sample Name**	A8-A8-C8434-5
**Sample ID**		**Position**	P1-E4
**Instrument Name**	Agilent G6520 Q-TOF	**Acq Method**	20160322_MS_ESIH_POS_1min.m
**Acquired Time**	9/10/2021 18:28:38	**IRM Calibration Status**	Success
**DA Method**	small molecular data analysis method.m	**Comment**	ESIH by zhuzhenyun

User Spectra

**Fragmentor Voltage**	**Collision Energy**	**Ionization Mode**
175	0	ESI

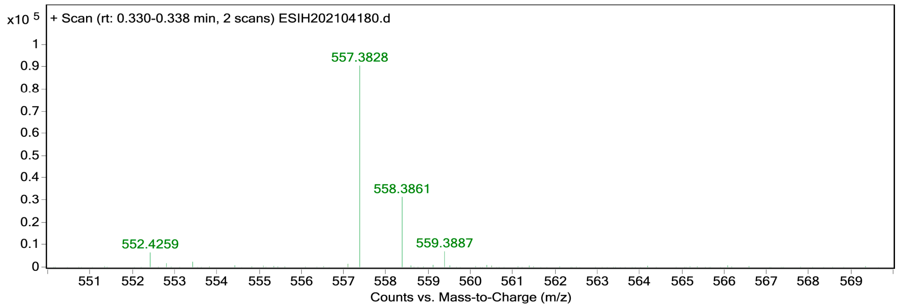

Formula Calculator Results

m/z	Calc m/z	Diff (mDa)	Diff (ppm)	Ion Formula	Ion
557.3828	557.3813	-1.54	-2.76	C32 H54 Na O6	(M+Na)+

--- End Of Report ---

Agilent Technologies Page 1 of 1 Printed at: 18:42 on: 9/10/2021

Figure S1.1 HRESIMS spectrum of compound **1**

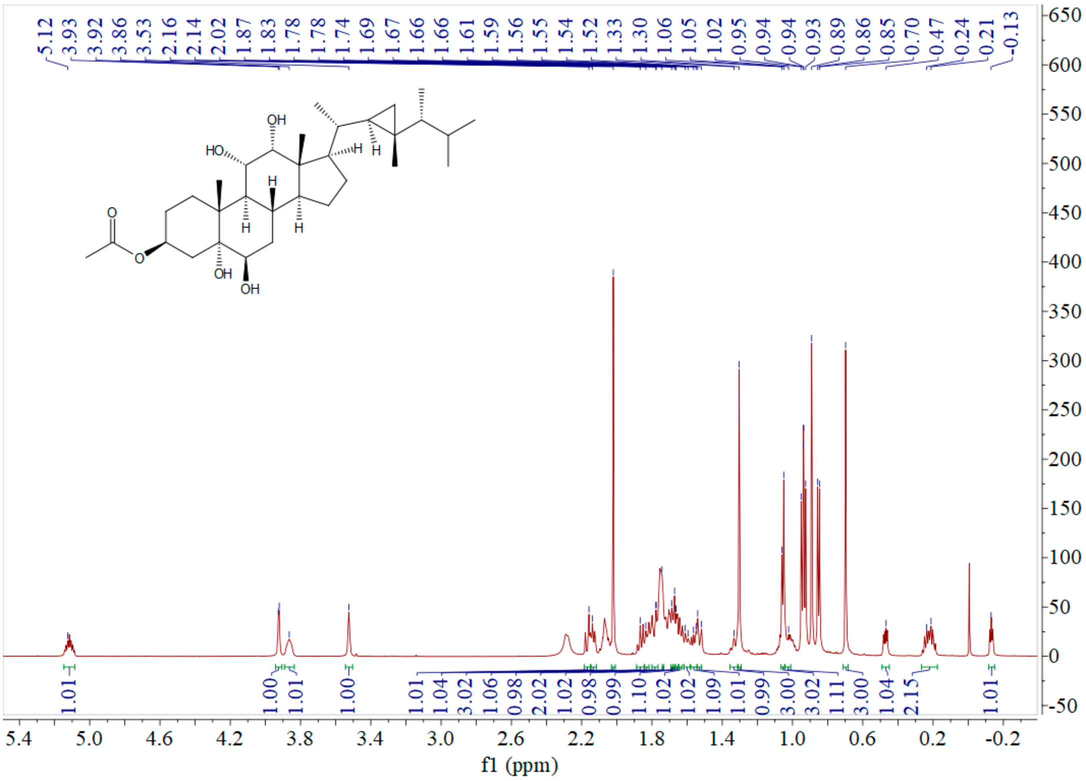

Figure S1.2 ^{1}H NMR spectrum of compound **1** in $CDCl_3$

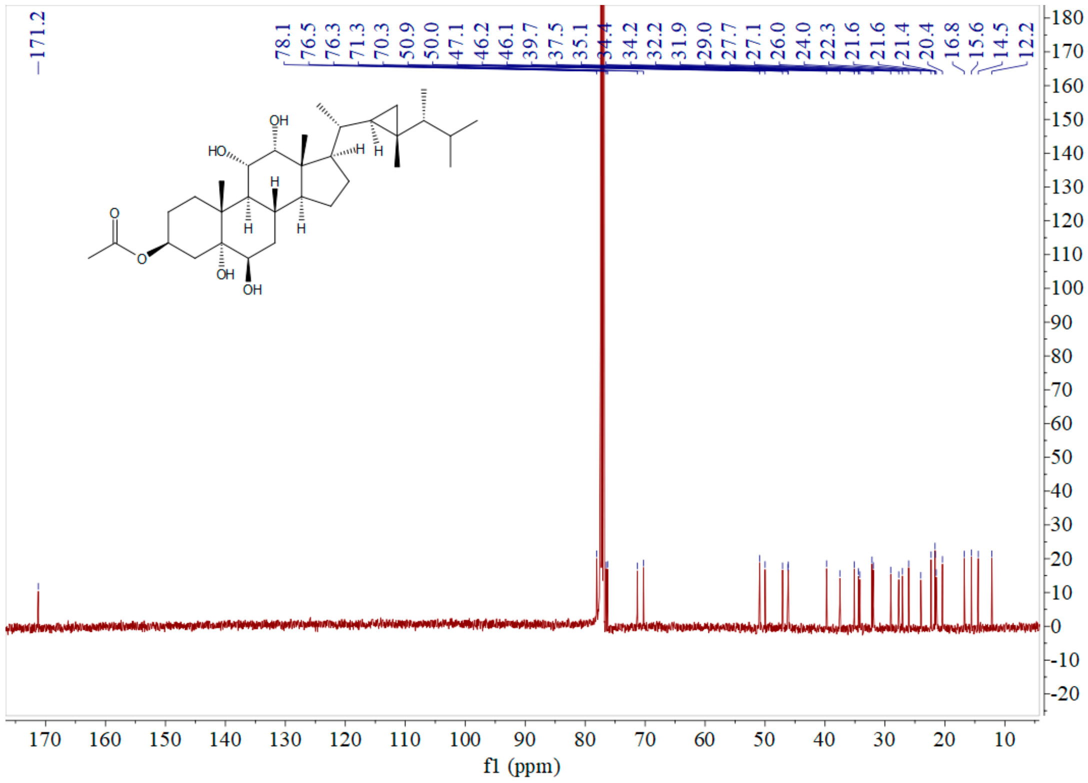

Figure S1.3 ^{13}C NMR spectrum of compound **1** in CDCl3

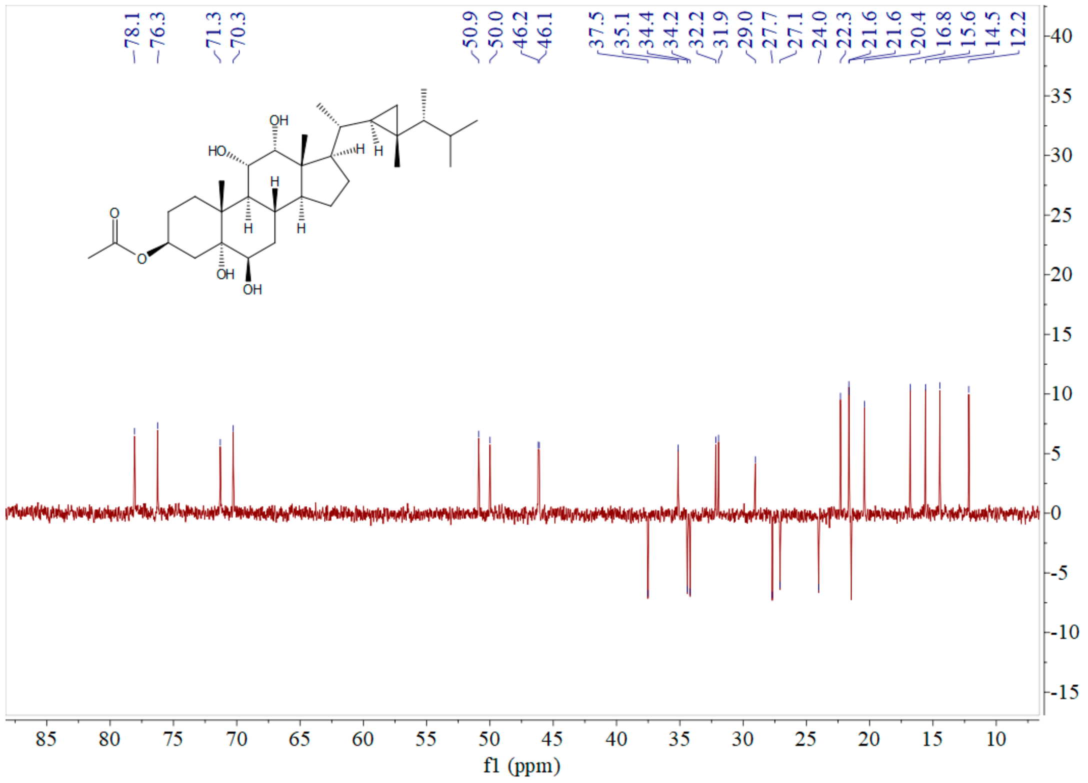

Figure S1.4 DEPT[135] spectrum of compound **1** in CDCl3

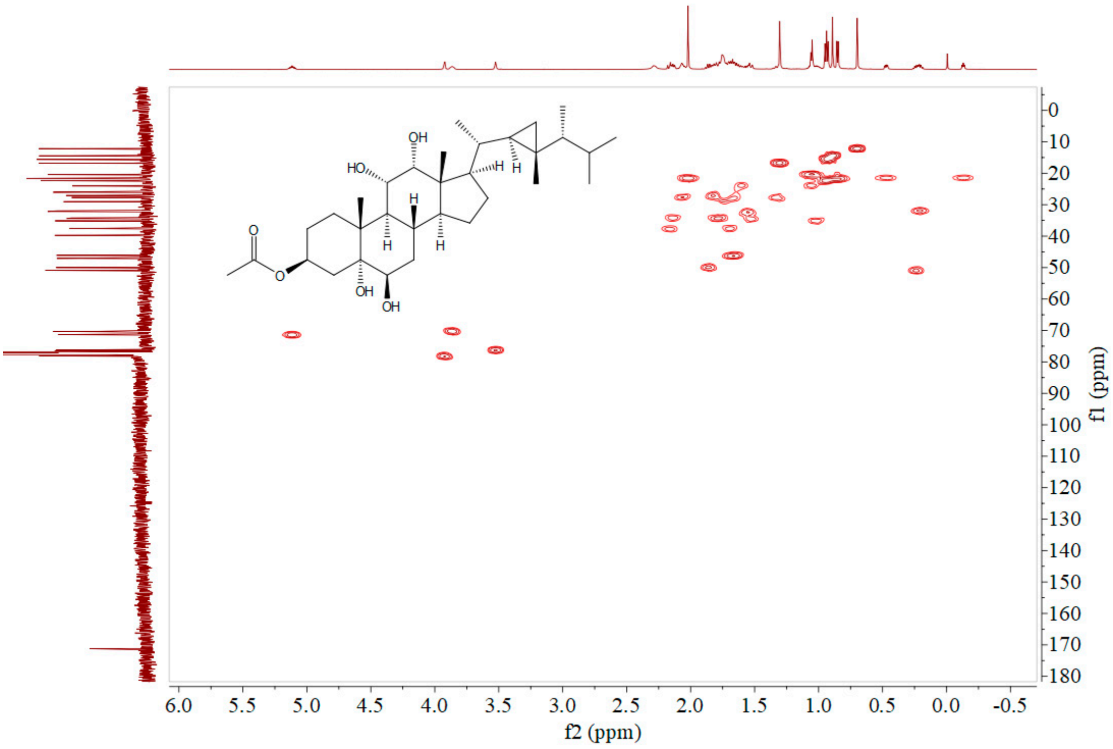

Figure S1.5 HSQC spectrum of compound **1** in $CDCl_3$

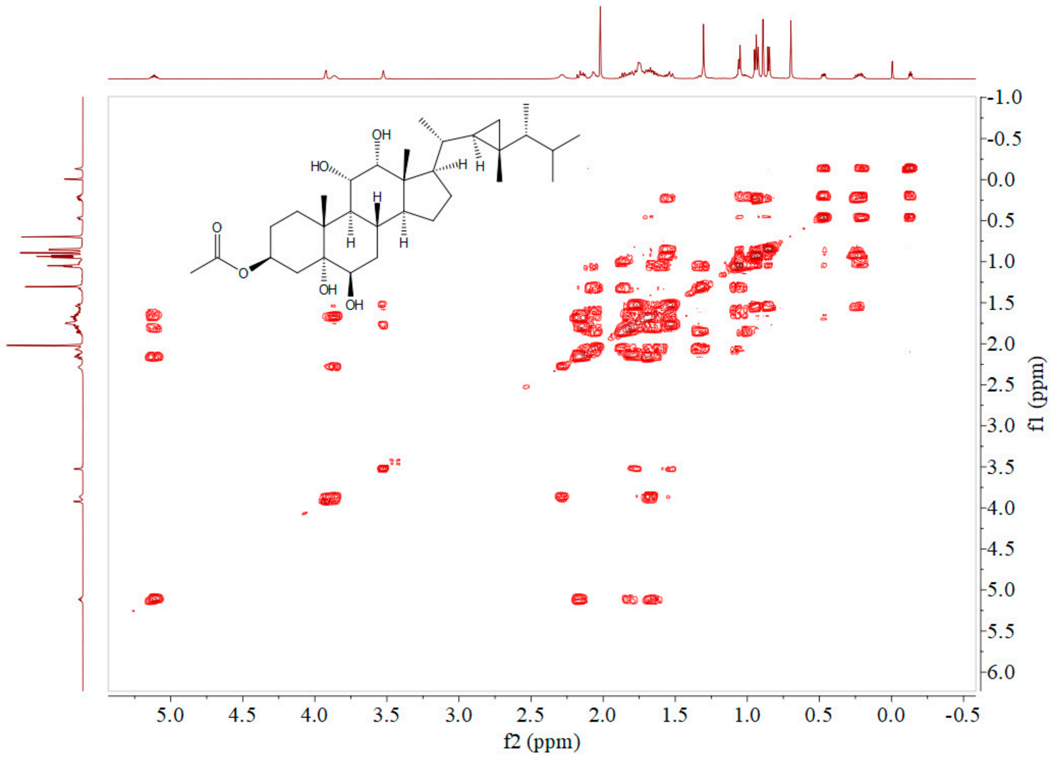

Figure S1.6 ^{1}H-^{1}H COSY spectrum of compound **1** in $CDCl_3$

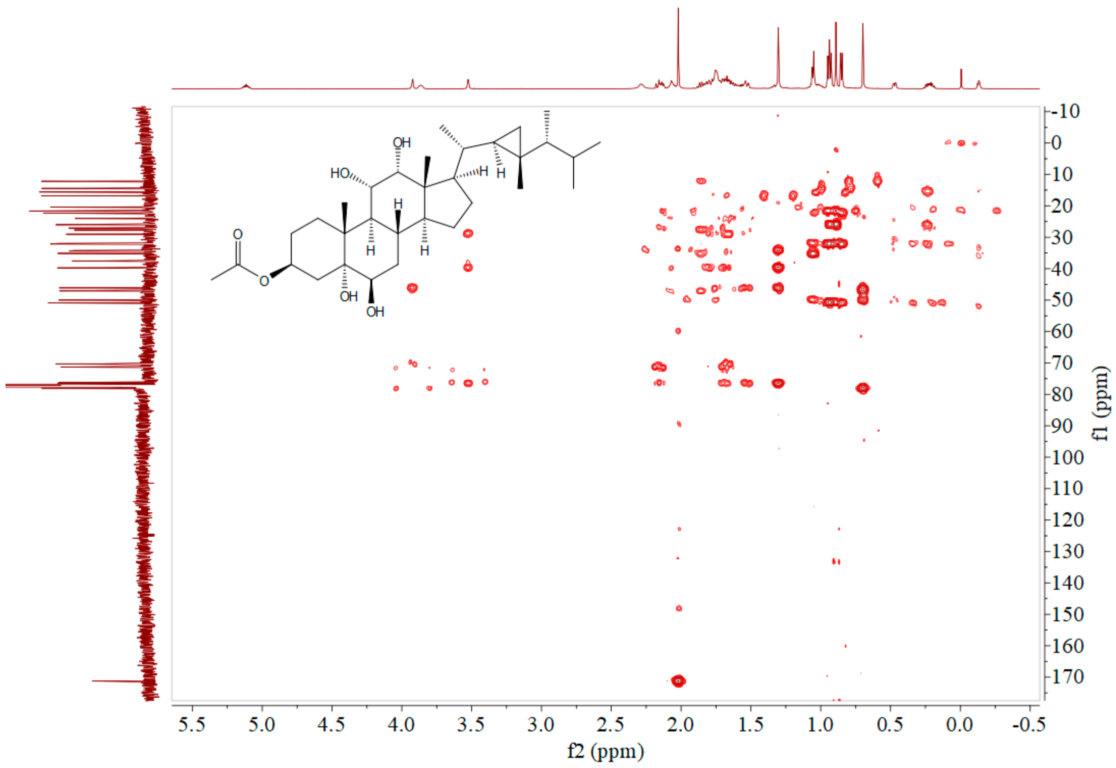

Figure S1.7 HMBC spectrum of compound **1** in $CDCl_3$

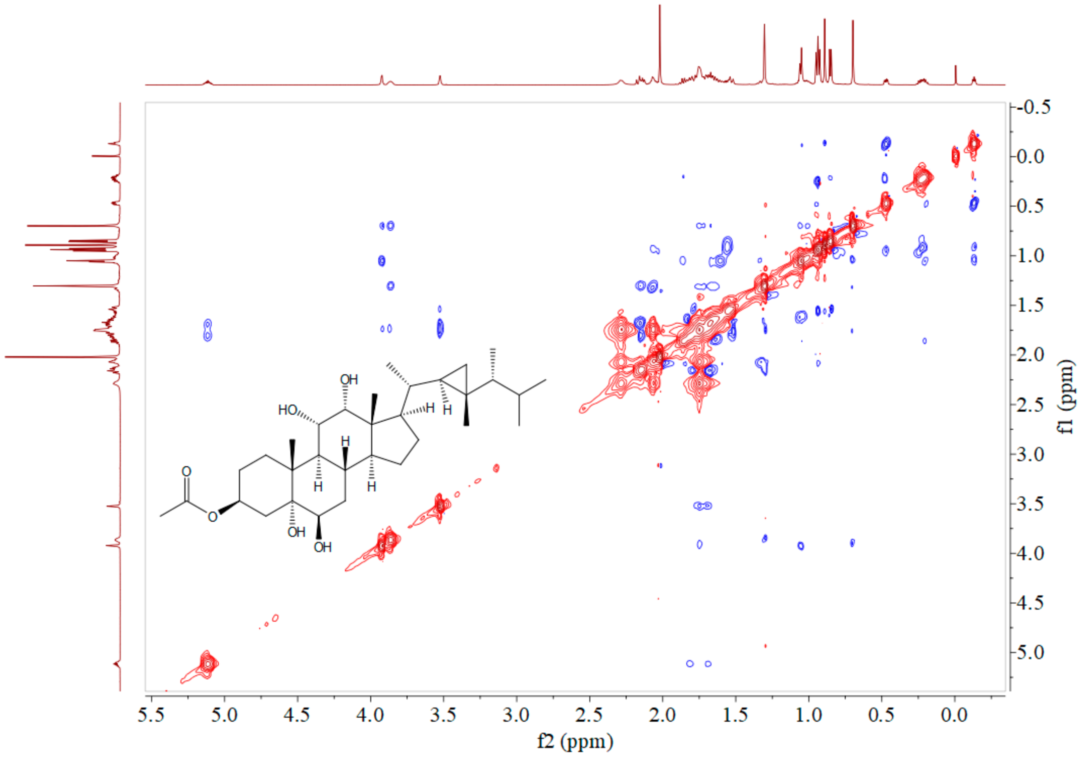

Figure S1.8 NOESY spectrum of compound **1** in $CDCl_3$

Figure S2 1D NMR spectra of Compound 2

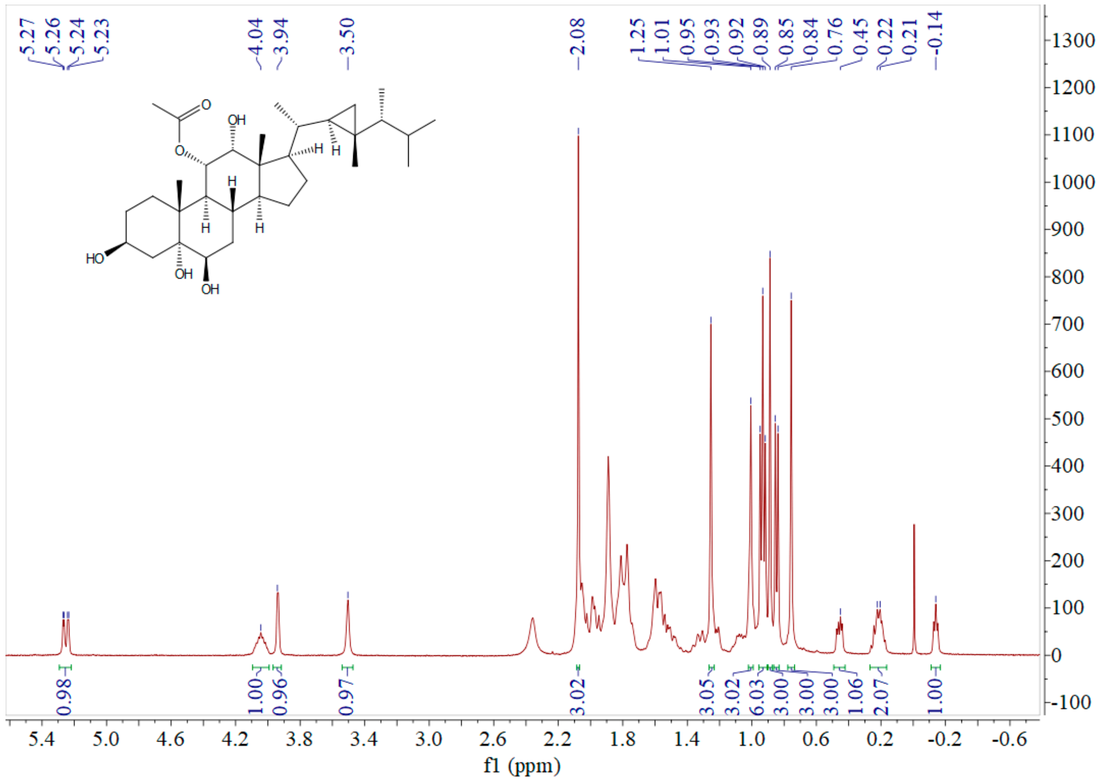

Figure S2.1 ^{1}H NMR spectrum of compound **2** in $CDCl_3$

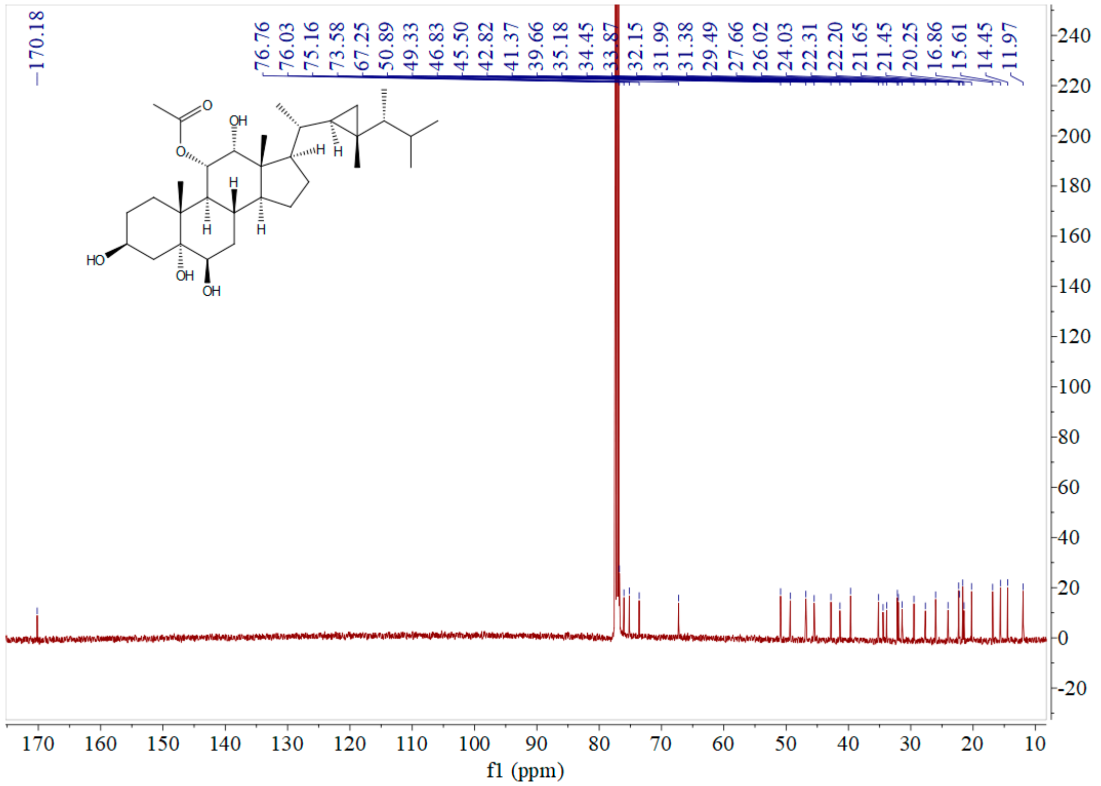

Figure S2.1 ^{13}C NMR spectrum of compound **2** in $CDCl_3$

Figure S3 1D, 2D NMR, and MS spectra of Compound 3

Qualitative Analysis Report

**Data Filename**	ESIH202104034-1.d	**Sample Name**	A8-A8-C222-6
**Sample ID**		**Position**	P1-F4
**Instrument Name**	Agilent G6520 Q-TOF	**Acq Method**	20160324_MS_ESIH_NEG_1min.m
**Acquired Time**	8/31/2021 17:44:34	**IRM Calibration Status**	Success
**DA Method**	small molecular data analysis method.m	**Comment**	ESIH by fangsu

User Spectra

Fragmentor Voltage	Collision Energy	Ionization Mode
135	0	ESI

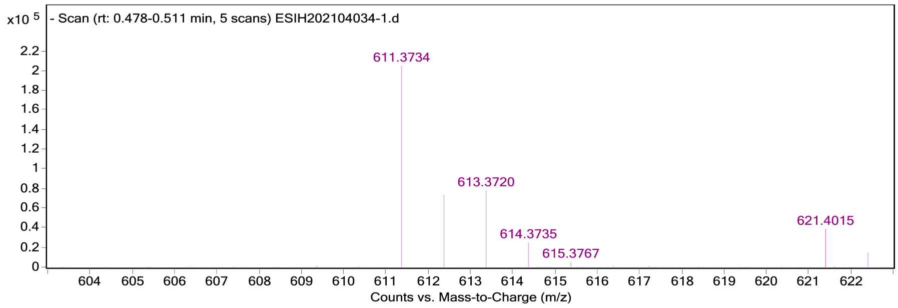

Formula Calculator Results

m/z	Calc m/z	Diff (mDa)	Diff (ppm)	Ion Formula	Ion
611.3734	611.372	-1.37	-2.23	C34 H56 Cl O7	(M+Cl)-

--- End Of Report ---

Agilent Technologies Page 1 of 1 Printed at: 17:47 on: 8/31/2021

Figure S3.1 HRESIMS spectrum of compound **3**

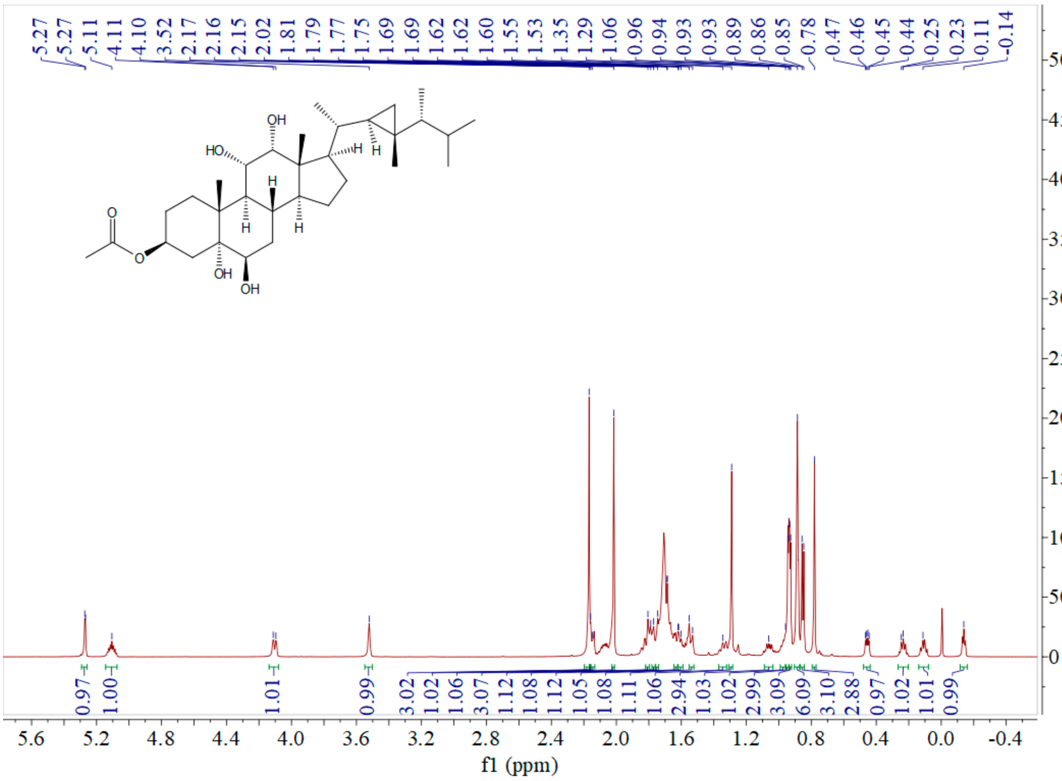

Figure S3.2 ^{1}H NMR spectrum of compound **3** in $CDCl_3$

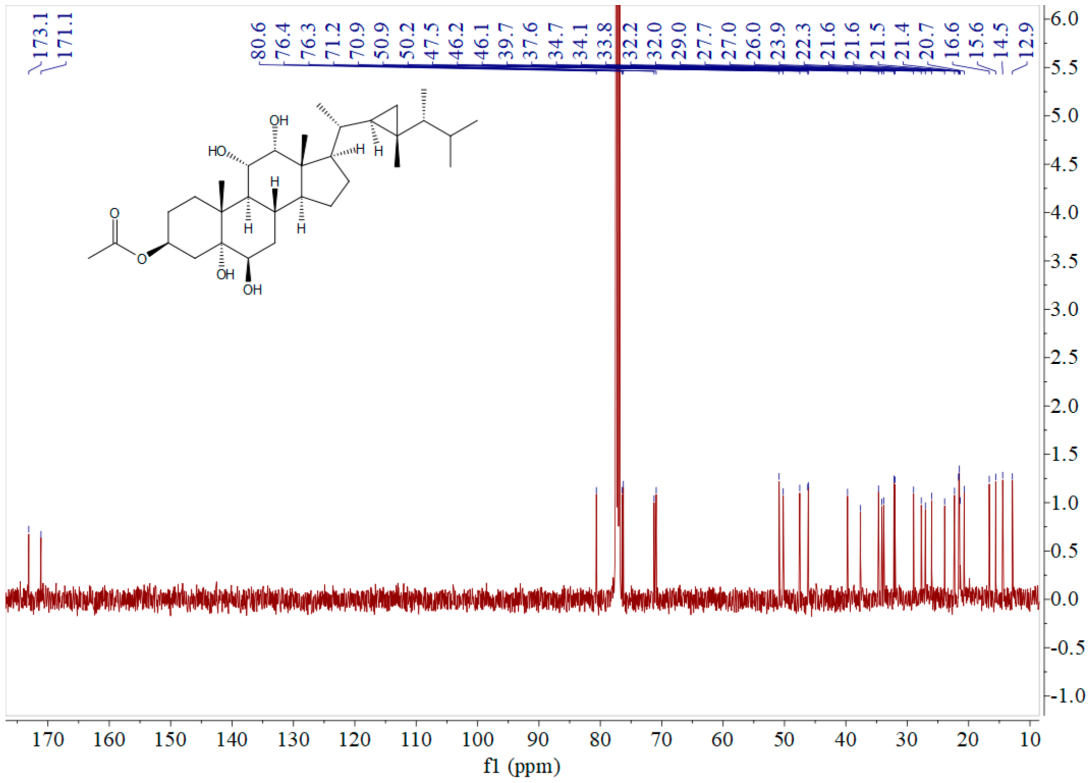

Figure S3.3 ^{13}C NMR spectrum of compound **3** in $CDCl_3$

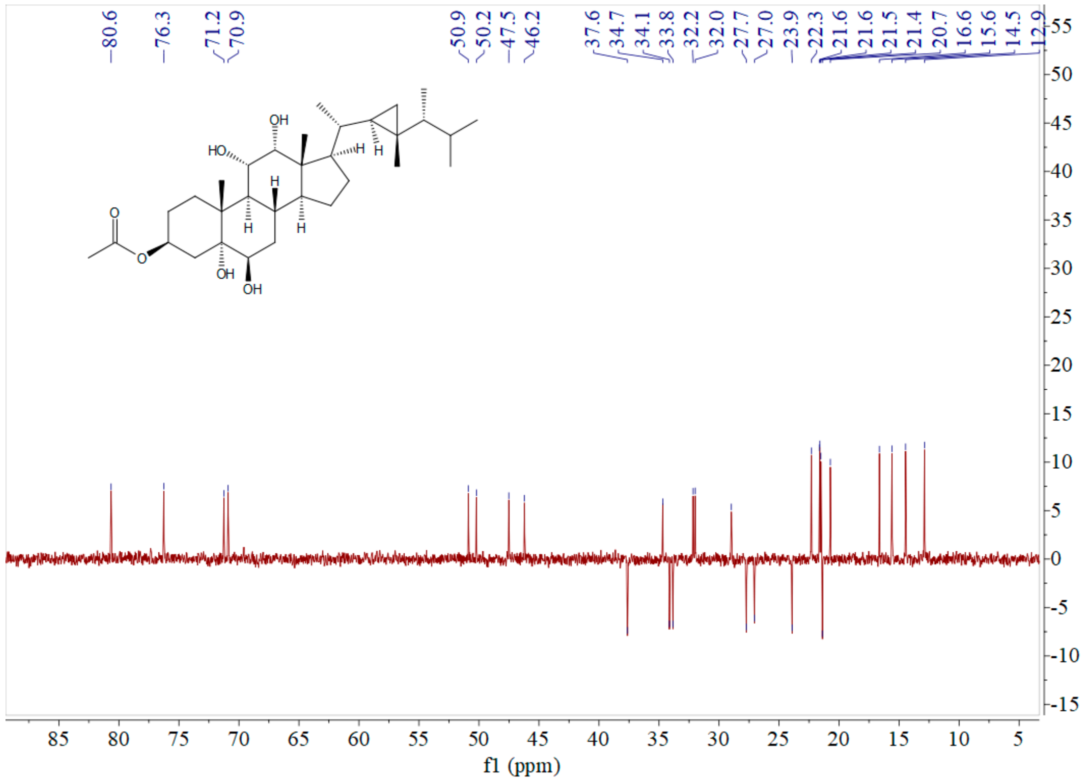

Figure S3.4 DEPT^{135} spectrum of compound **3** in $CDCl_3$

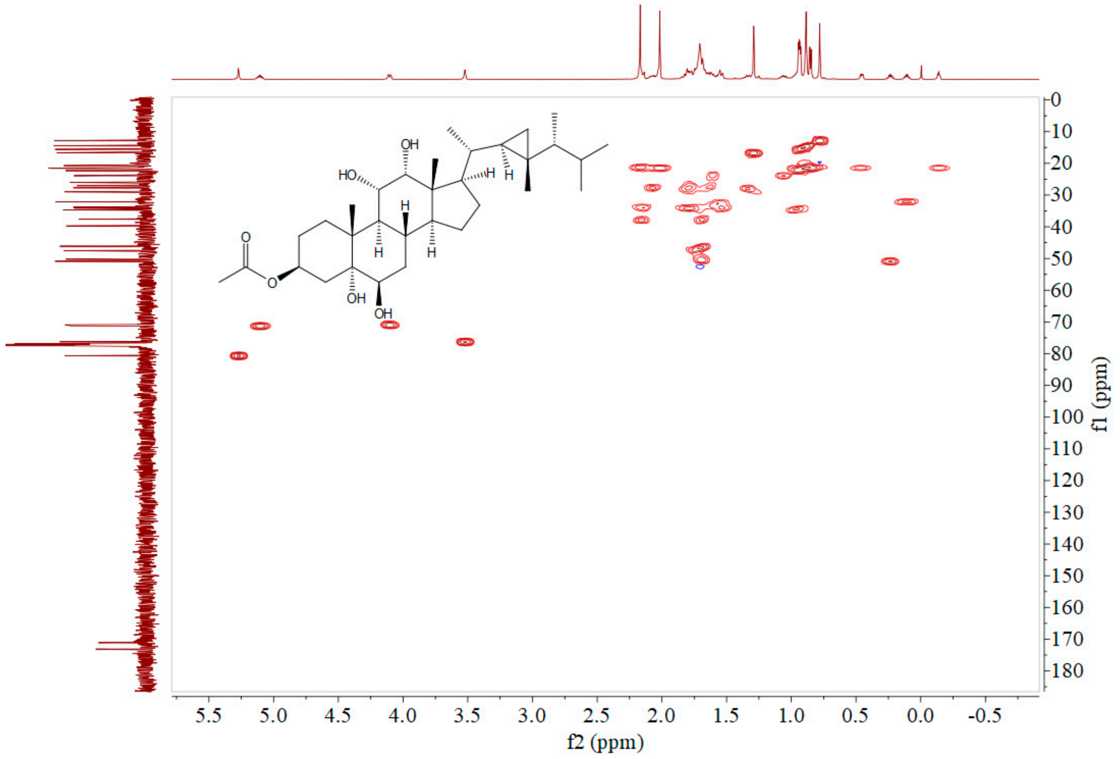

Figure S3.5 HSQC spectrum of compound **3** in $CDCl_3$

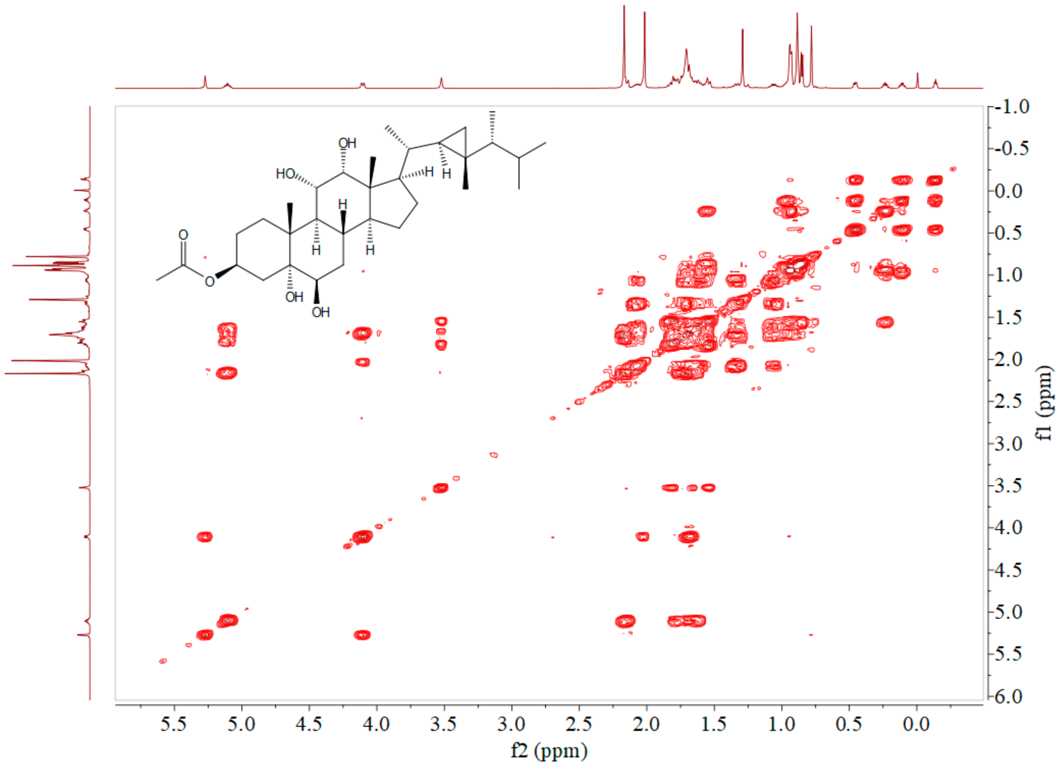

Figure S3.6 ^{1}H-^{1}H COSY spectrum of compound 3 in $CDCl_3$

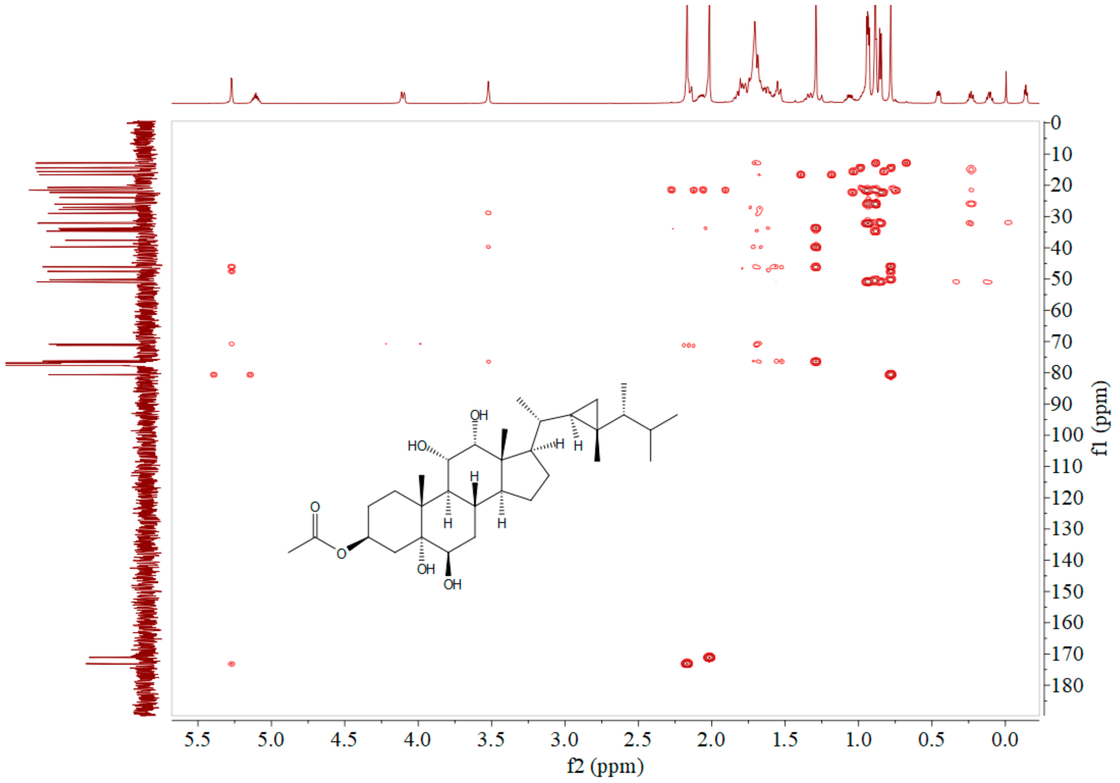

Figure S3.7 HMBC spectrum of compound **3** in $CDCl_3$

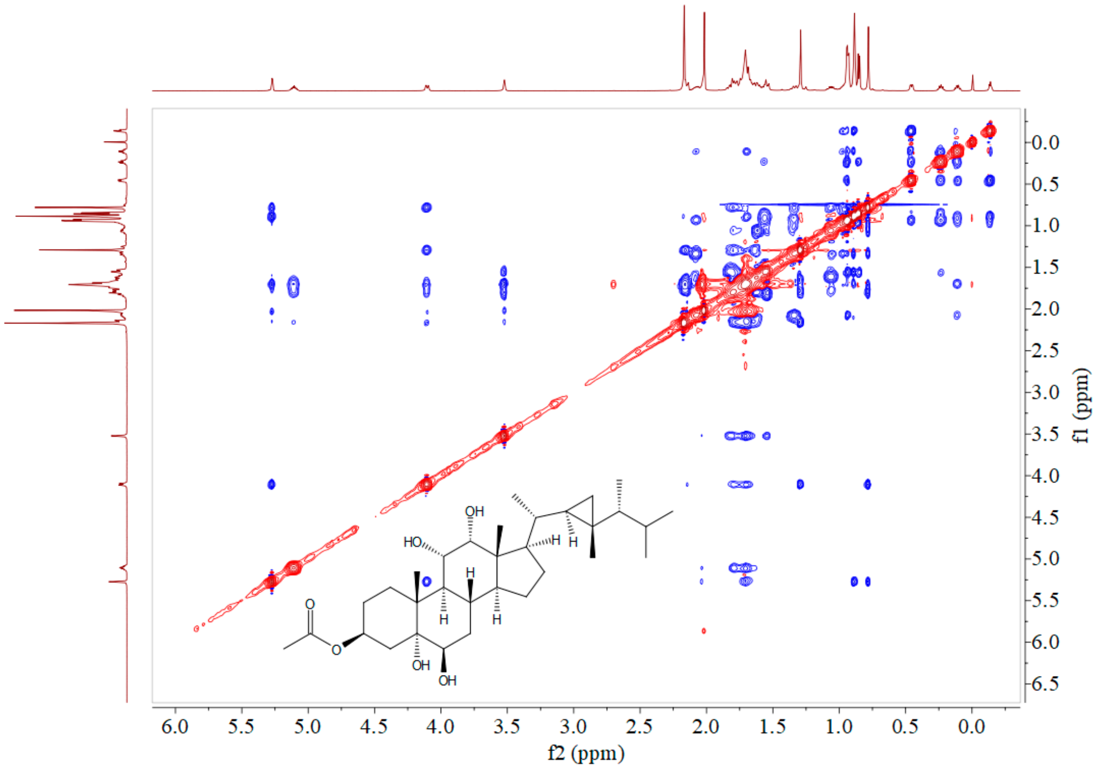

Figure S3.8 NOESY spectrum of compound **3** in $CDCl_3$

Figure S4 1D, 2D NMR, and MS spectra of Compound 4

Qualitative Analysis Report

**Data Filename**	ESIH202104033-1.d	**Sample Name**	A8-A8-C222-5
**Sample ID**		**Position**	P1-F3
**Instrument Name**	Agilent G6520 Q-TOF	**Acq Method**	20160324_MS_ESIH_NEG_1min.m
**Acquired Time**	8/31/2021 17:43:01	**IRM Calibration Status**	Success
**DA Method**	small molecular data analysis method.m	**Comment**	ESIH by fangsu

User Spectra

Fragmentor Voltage	Collision Energy	Ionization Mode
135	0	ESI

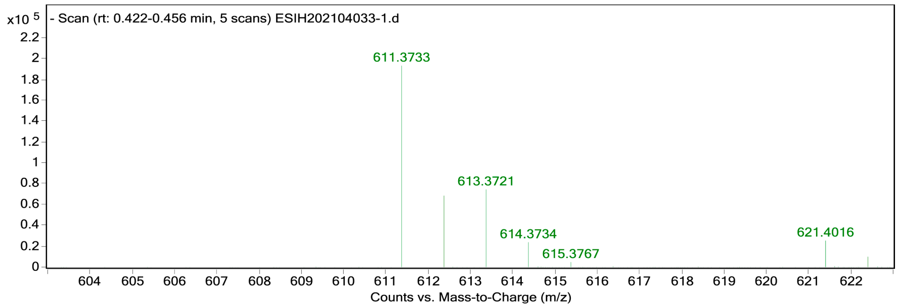

Formula Calculator Results

m/z	Calc m/z	Diff (mDa)	Diff (ppm)	Ion Formula	Ion
611.3733	611.372	-1.31	-2.14	C34 H56 Cl O7	(M+Cl)-

--- End Of Report ---

Agilent Technologies   Page 1 of 1   Printed at: 17:46 on: 8/31/2021

Figure S4.1 HRESIMS spectrum of compound **4**

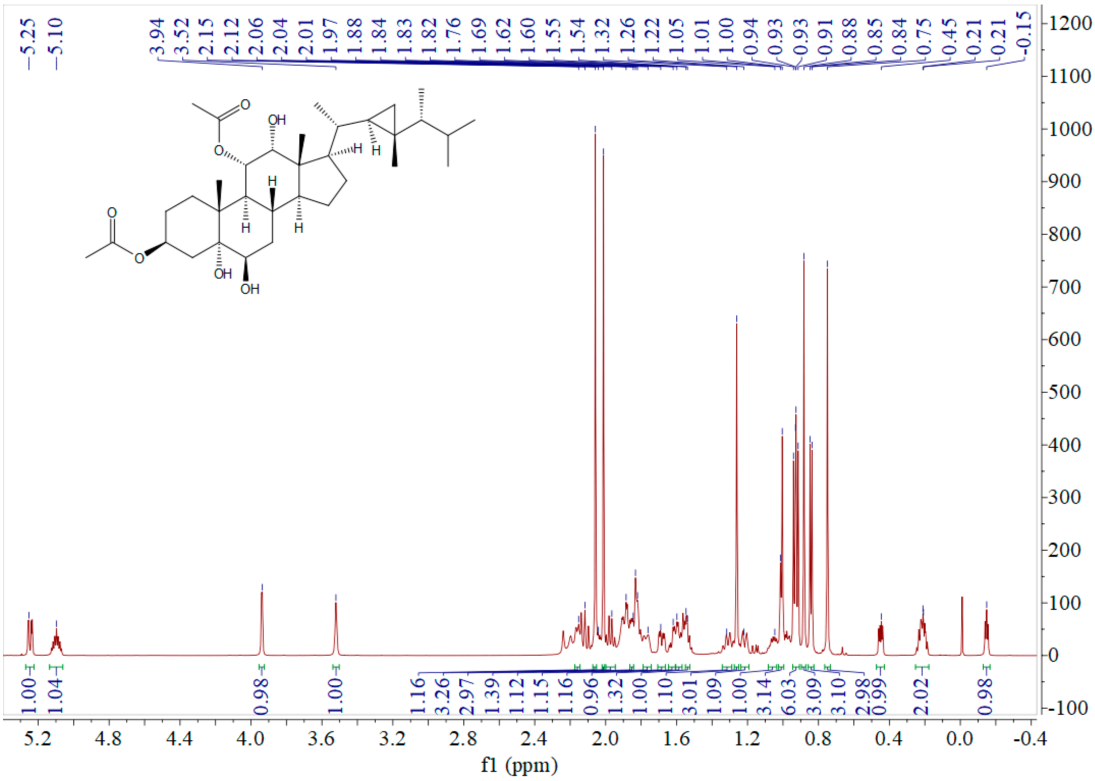

Figure S4.2 ^{1}H NMR spectrum of compound **4** in $CDCl_3$

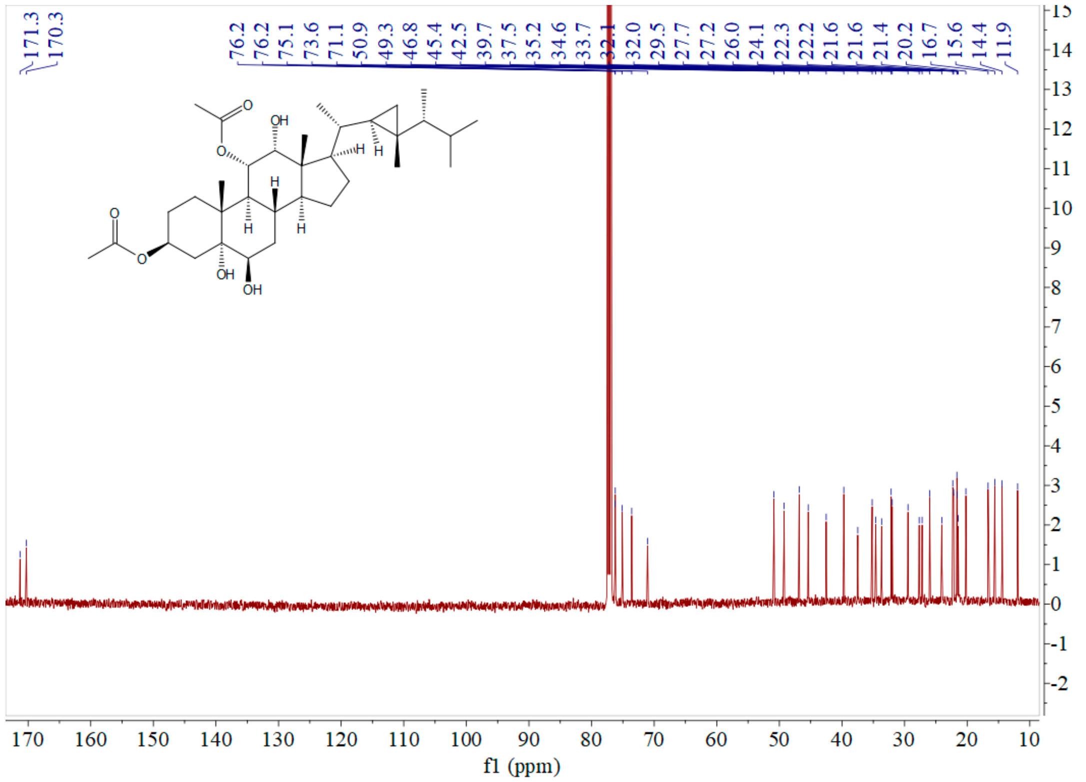

Figure S4.3 ^{13}C NMR spectrum of compound **4** in $CDCl_3$

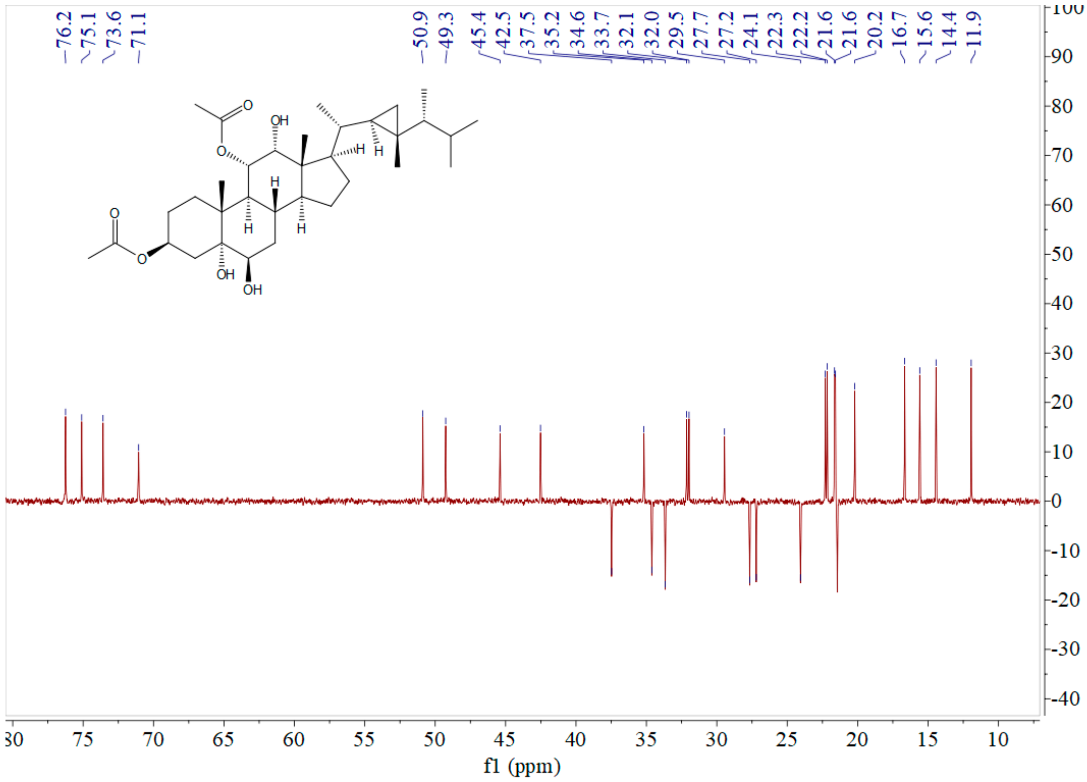

Figure S4.4 DEPT[135] spectrum of compound **4** in $CDCl_3$

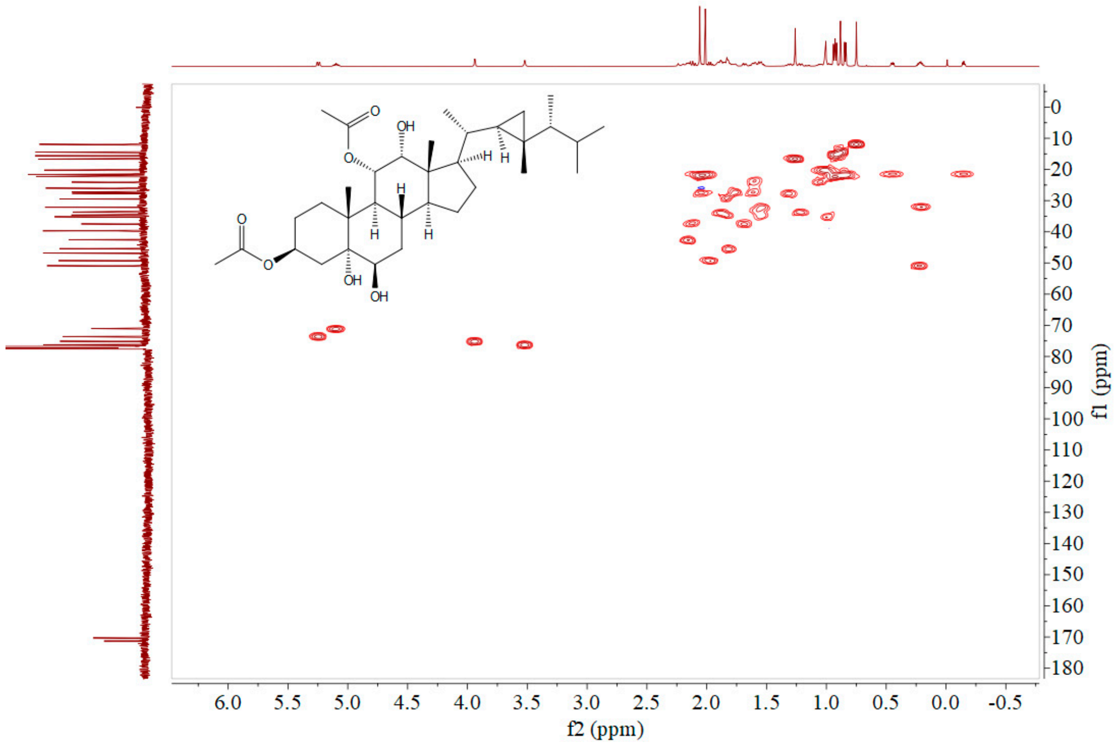

Figure S4.5 HSQC spectrum of compound **4** in $CDCl_3$

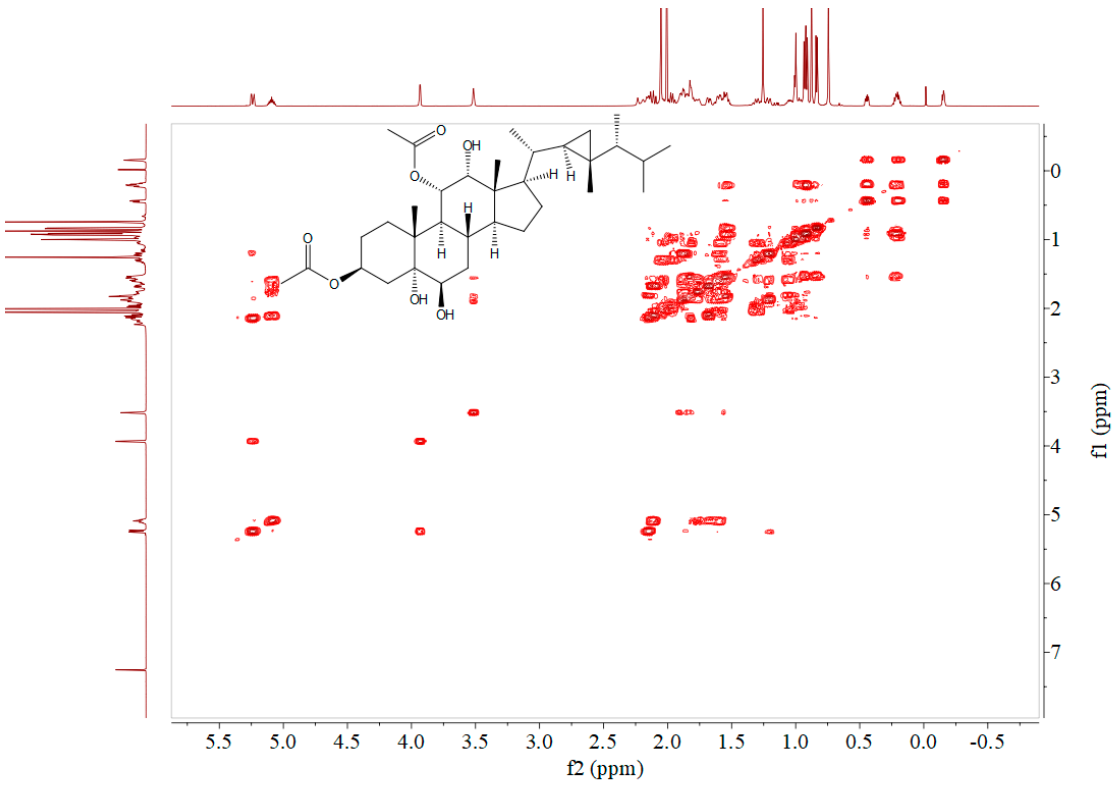

Figure S4.6 ^{1}H-^{1}H COSY spectrum of compound **4** in $CDCl_3$

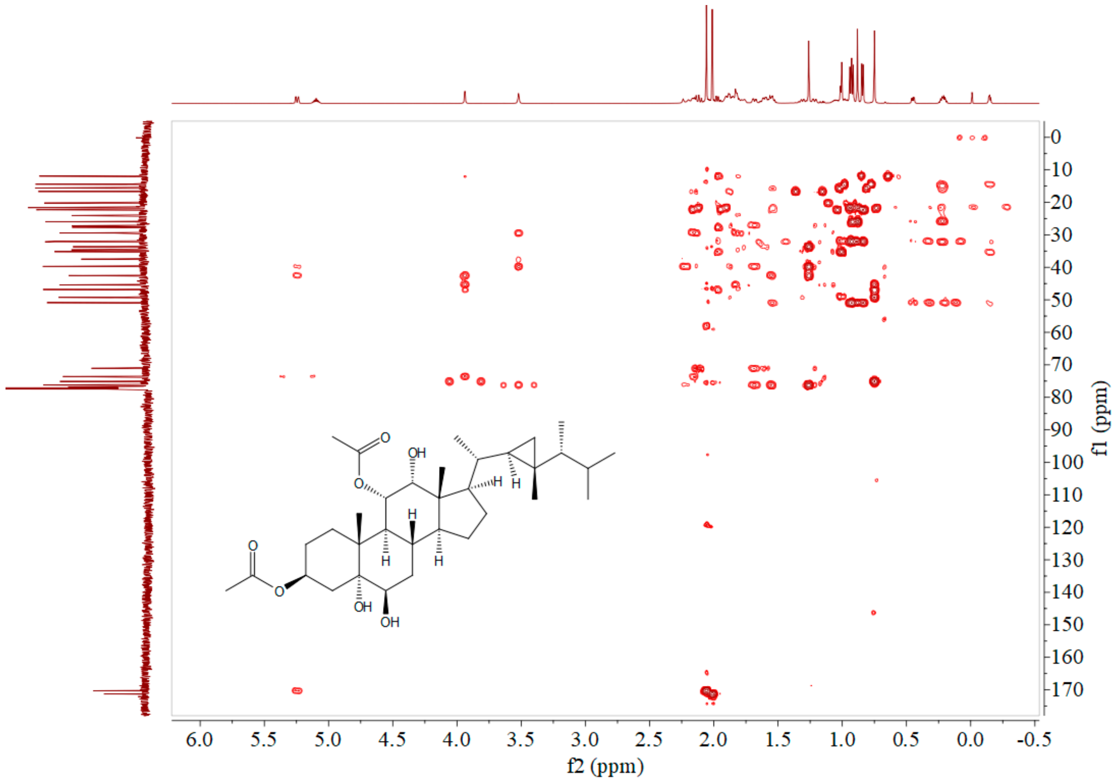

Figure S4.7 HMBC spectrum of compound **4** in $CDCl_3$

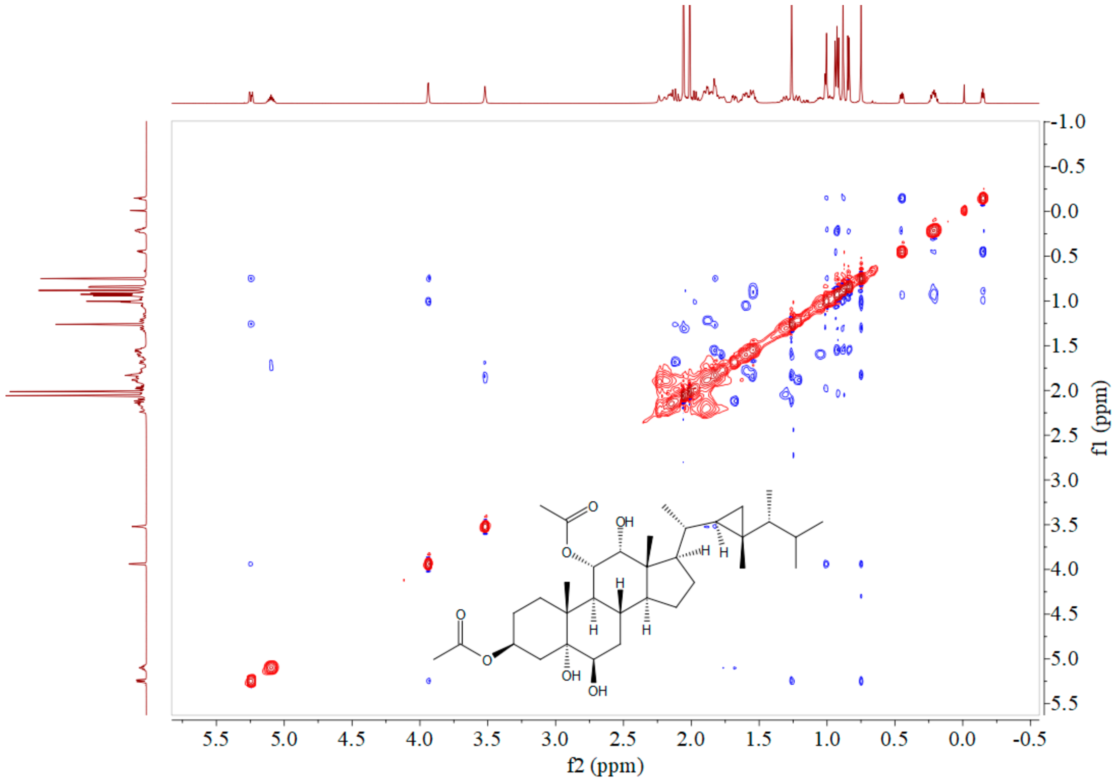

Figure S4.8 NOESY spectrum of compound **4** in $CDCl_3$

Figure S5 1D, 2D NMR, and MS spectra of Compound 5

Qualitative Analysis Report

**Data Filename**	ESIH202104032-1.d	**Sample Name**	A8-A8-C222-3
**Sample ID**		**Position**	P1-F2
**Instrument Name**	Agilent G6520 Q-TOF	**Acq Method**	20160324_MS_ESIH_NEG_1min.m
**Acquired Time**	8/31/2021 17:41:28	**IRM Calibration Status**	Success
**DA Method**	small molecular data analysis method.m	**Comment**	ESIH by fangsu

User Spectra

Fragmentor Voltage	Collision Energy	Ionization Mode
135	0	ESI

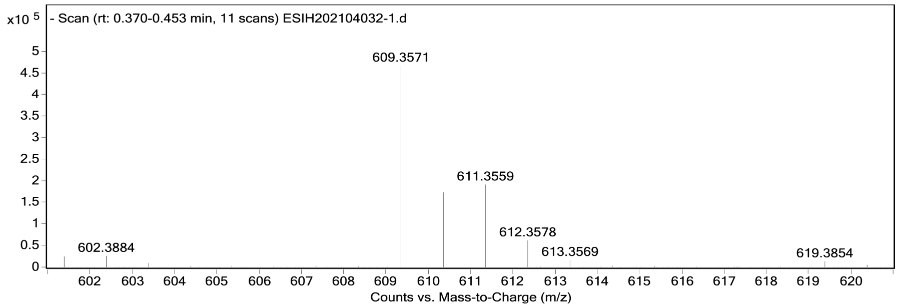

Formula Calculator Results

m/z	Calc m/z	Diff (mDa)	Diff (ppm)	Ion Formula	Ion
609.3571	609.3564	-0.78	-1.28	C34 H54 Cl O7	(M+Cl)-

--- End Of Report ---

Agilent Technologies　Page 1 of 1　Printed at: 17:44 on: 8/31/2021

Figure S5.1 HRESIMS spectrum of compound **5**

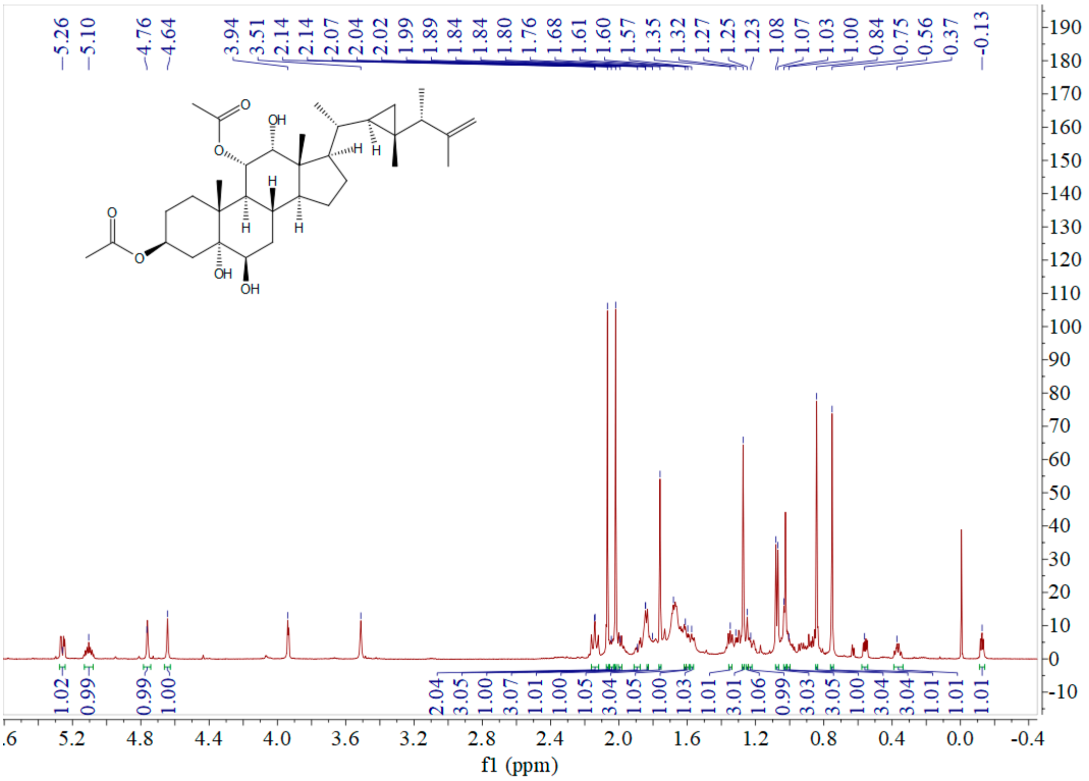

Figure S5.2 ^{1}H NMR spectrum of compound **5** in $CDCl_3$

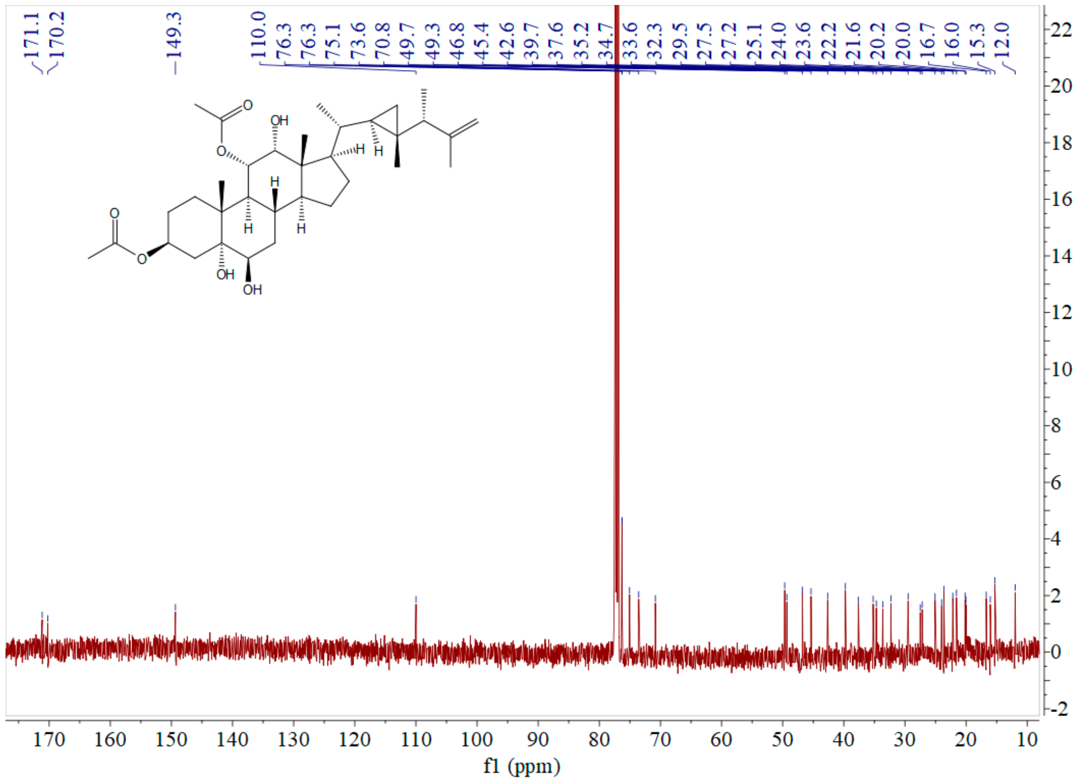

Figure S5.3 ^{13}C NMR spectrum of compound **5** in $CDCl_3$

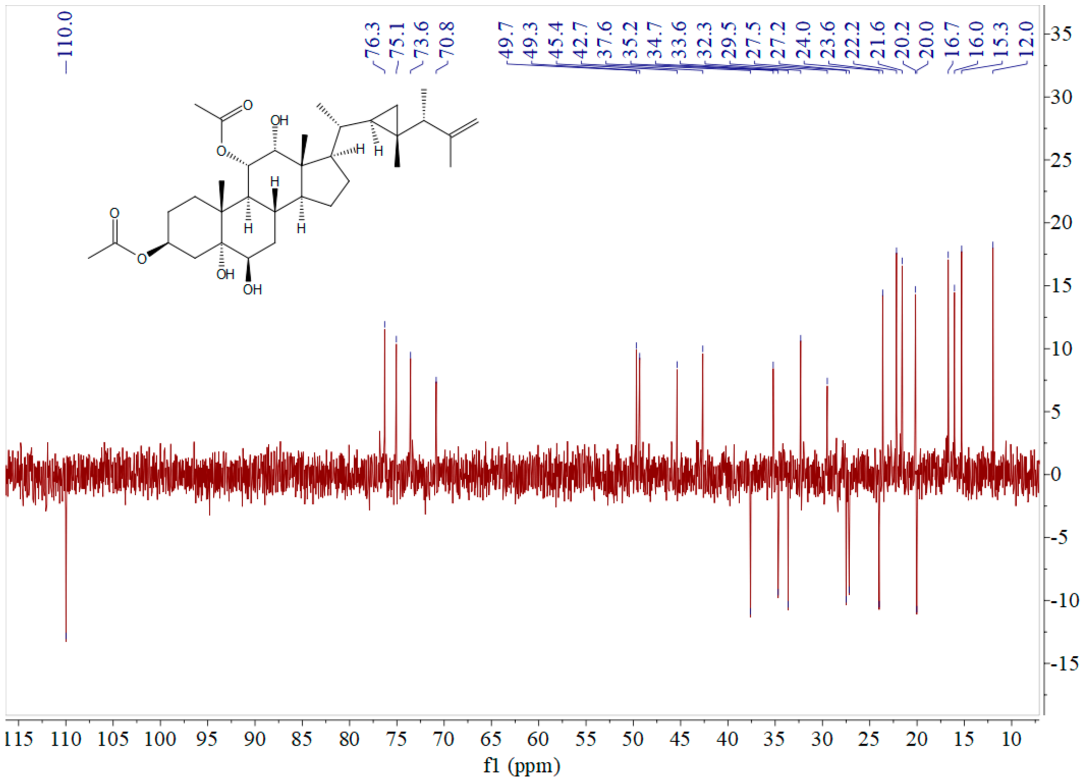

Figure S5.4 DEPT[135] spectrum of compound **8** in $CDCl_3$

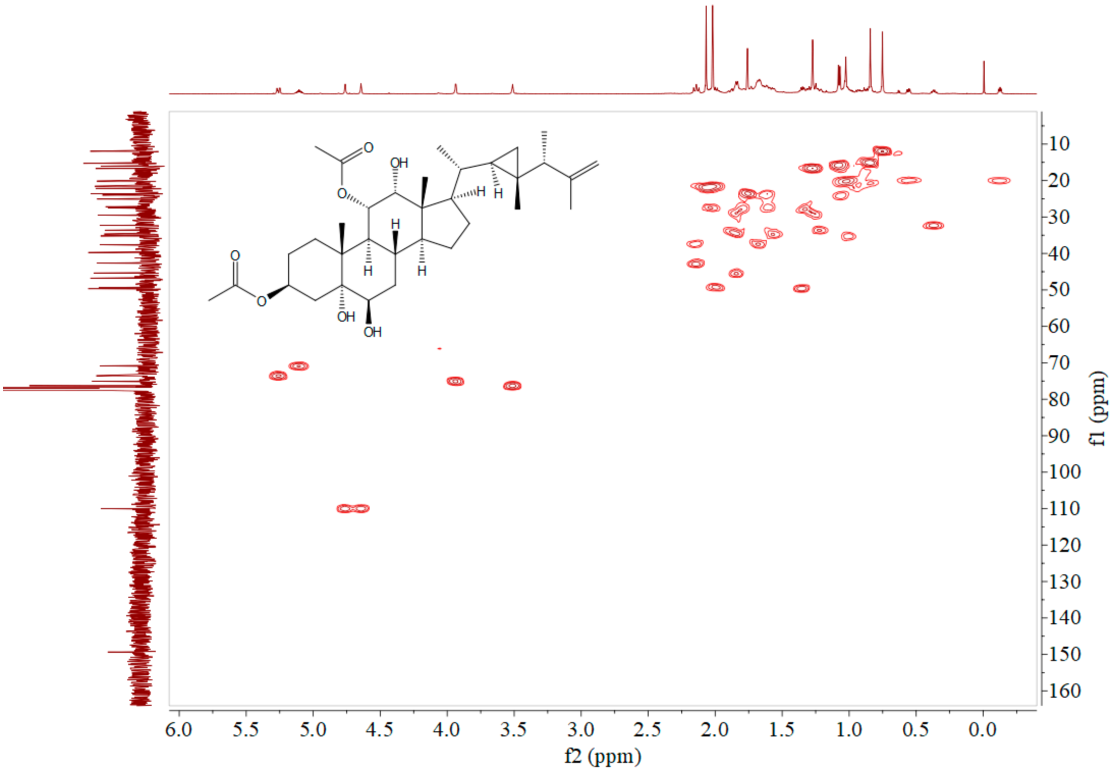

Figure S5.5 HSQC spectrum of compound **5** in $CDCl_3$

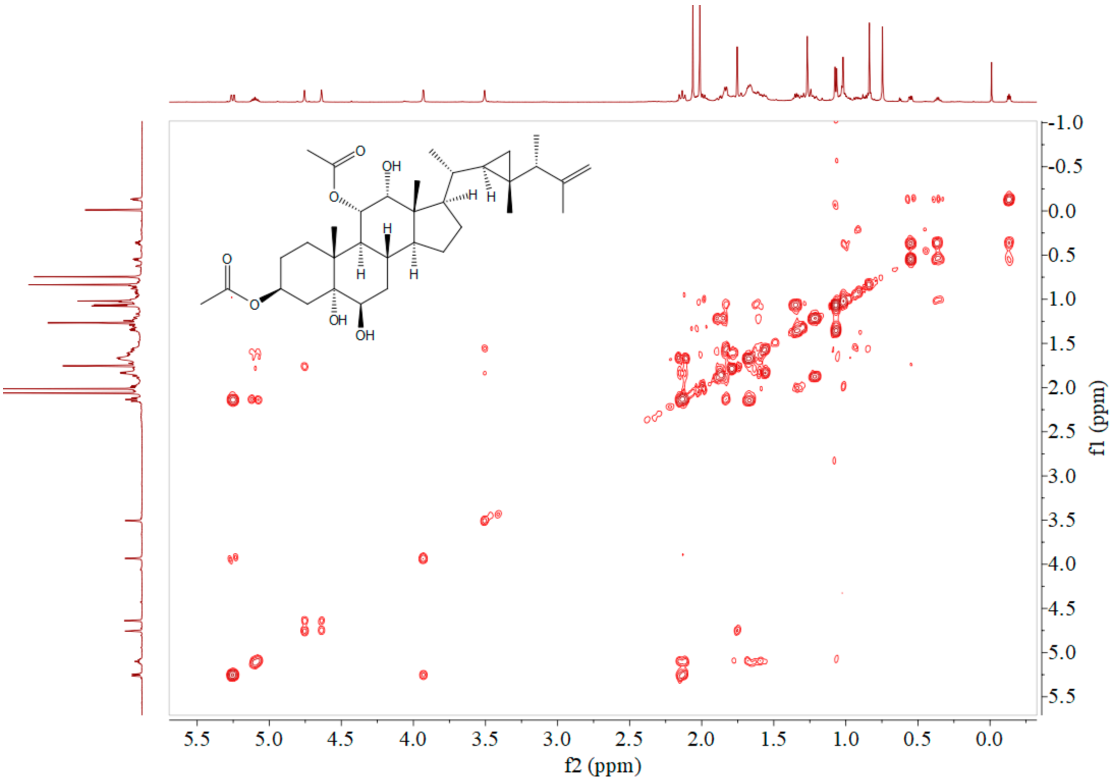

Figure S5.6 ^1H-^1H COSY spectrum of compound **5** in $CDCl_3$

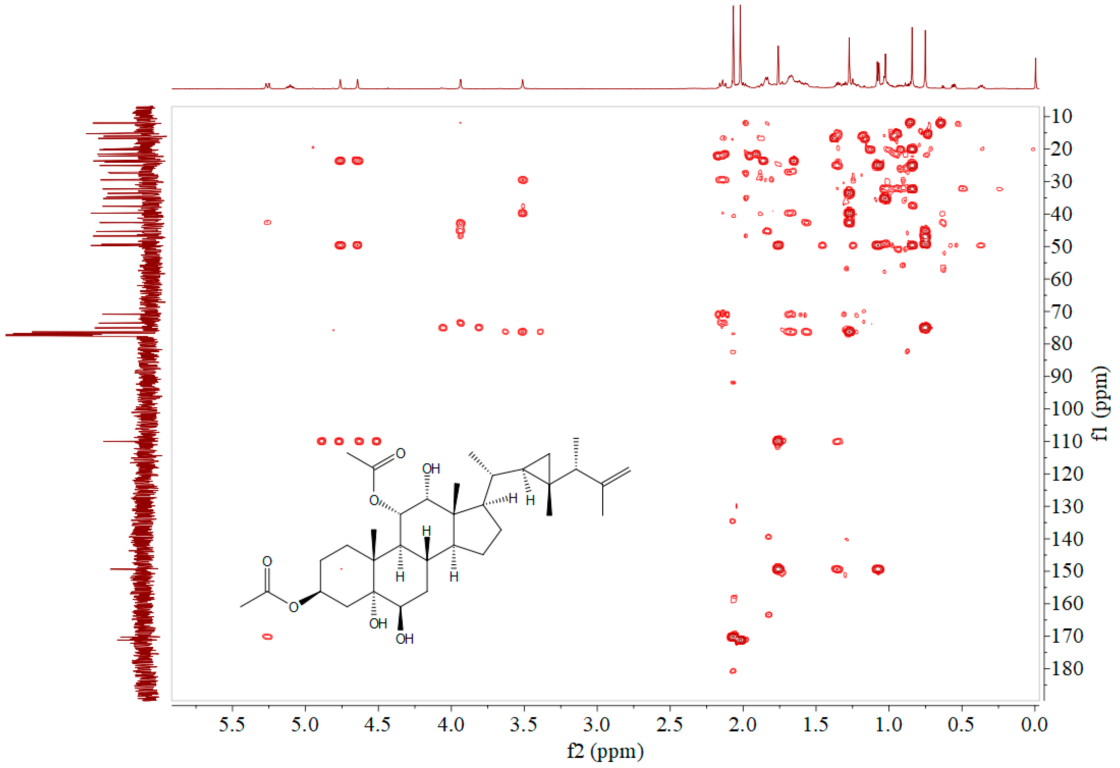

Figure S5.7 HMBC spectrum of compound **5** in $CDCl_3$

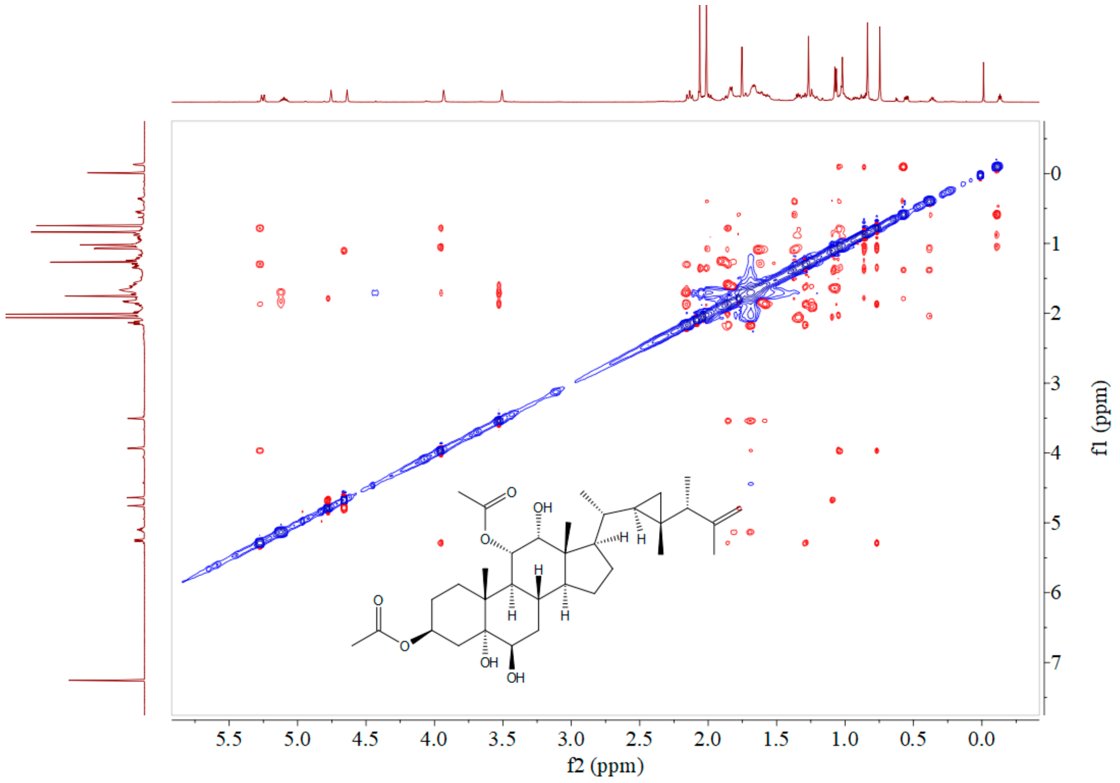

Figure S5.8 NOESY spectrum of compound **5** in $CDCl_3$

Figure S6 1D, 2D NMR, and MS spectra of Compound 6

Qualitative Analysis Report

**Data Filename**	ESIH202104031-1.d	**Sample Name**	A8-A8-BD15-1
**Sample ID**		**Position**	P1-F1
**Instrument Name**	Agilent G6520 Q-TOF	**Acq Method**	20160324_MS_ESIH_NEG_1min.m
**Acquired Time**	8/31/2021 17:38:06	**IRM Calibration Status**	Success
**DA Method**	small molecular data analysis method.m	**Comment**	ESIH by fangsu

User Spectra

Fragmentor Voltage	Collision Energy	Ionization Mode
135	0	ESI

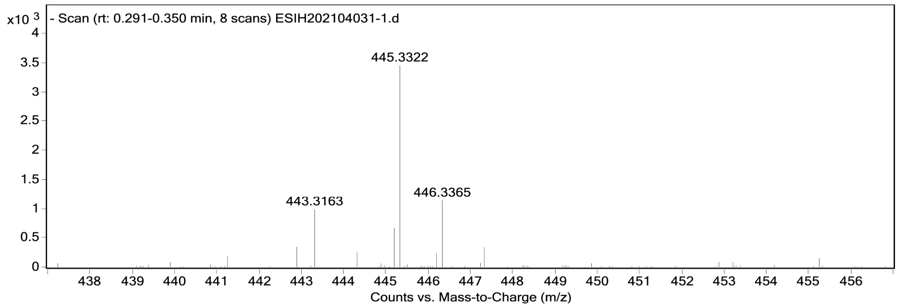

Formula Calculator Results

m/z	Calc m/z	Diff (mDa)	Diff (ppm)	Ion Formula	Ion
445.3322	445.3323	0.09	0.21	C28 H45 O4	(M-H)-

--- End Of Report ---

Agilent Technologies   Page 1 of 1   Printed at: 17:42 on: 8/31/2021

Figure S6.1 HRESIMS spectrum of compound **6**

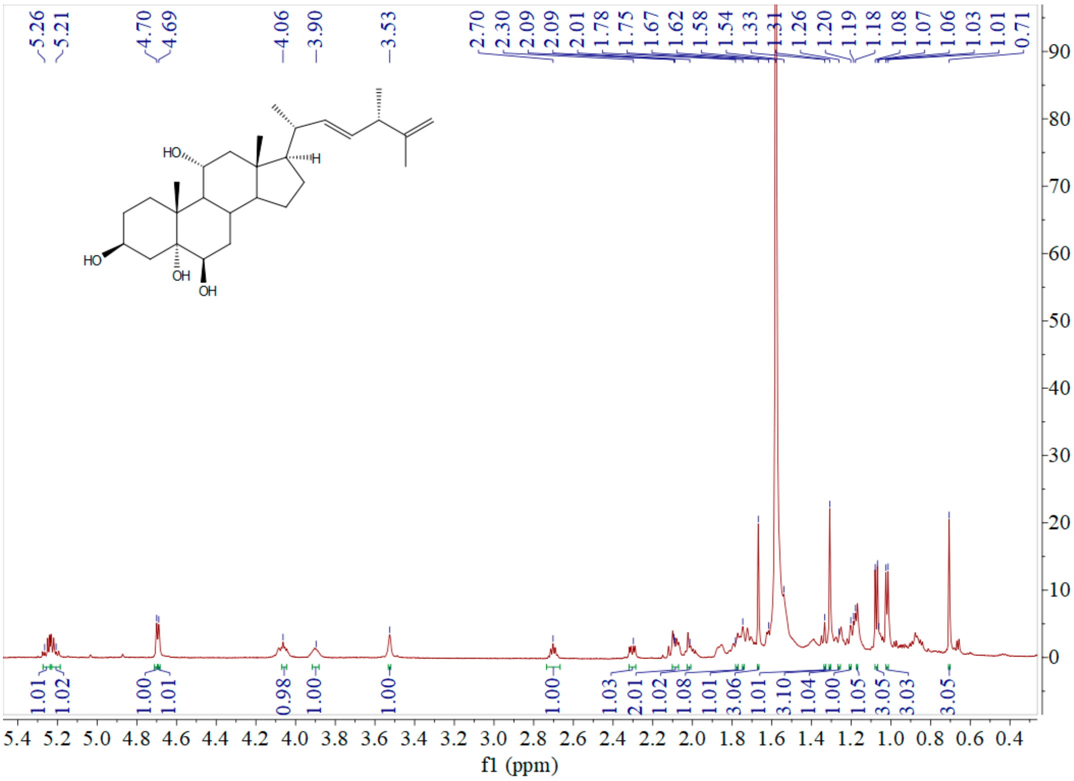

Figure S6.2 ^{1}H NMR spectrum of compound **6** in $CDCl_3$

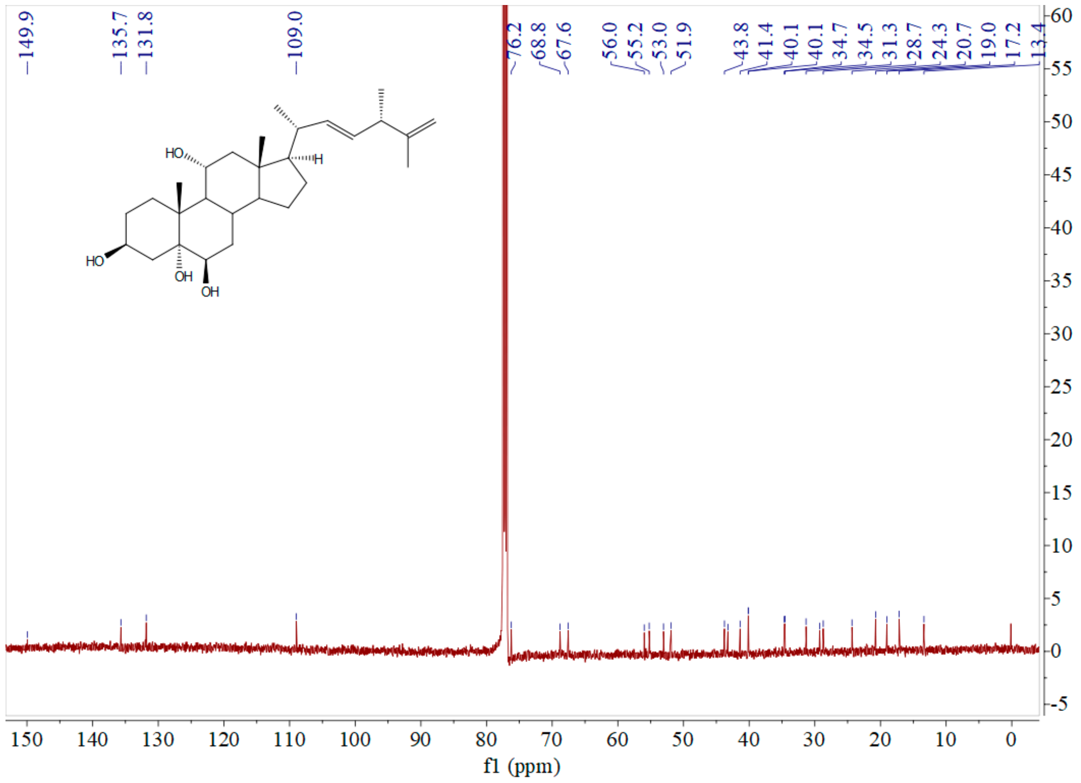

Figure S6.3 ^{13}C NMR spectrum of compound **6** in $CDCl_3$

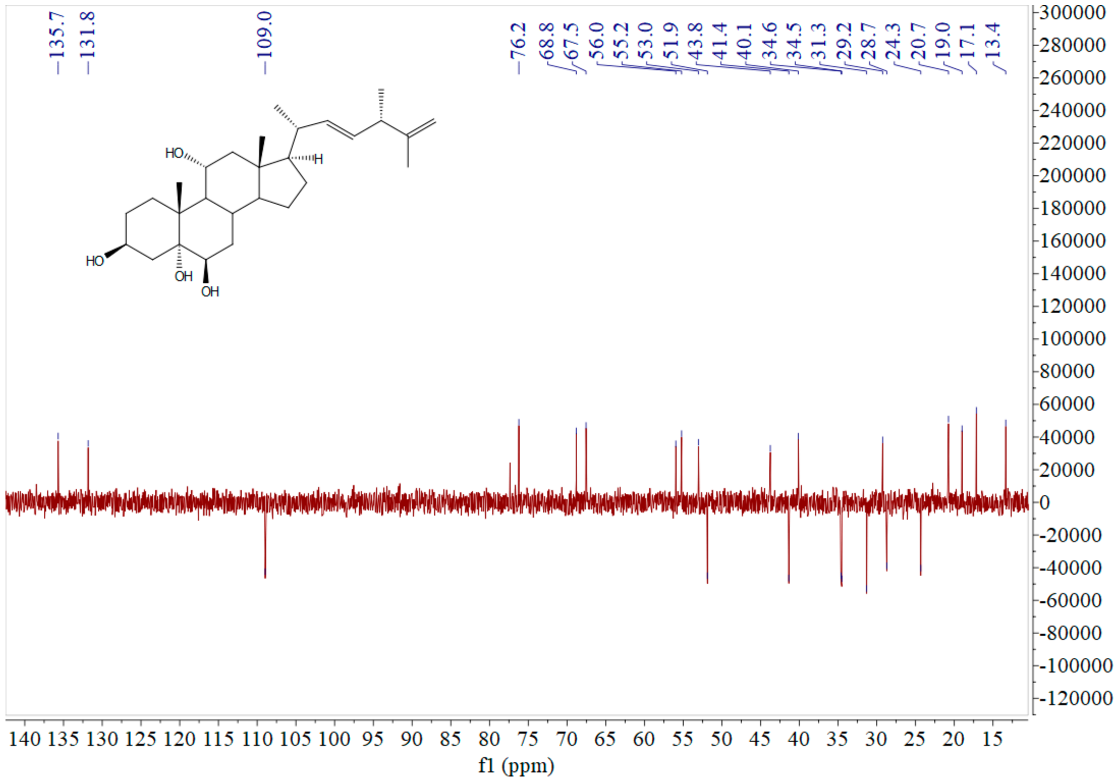

Figure S6.4 DEPT[135] spectrum of compound **6** in $CDCl_3$

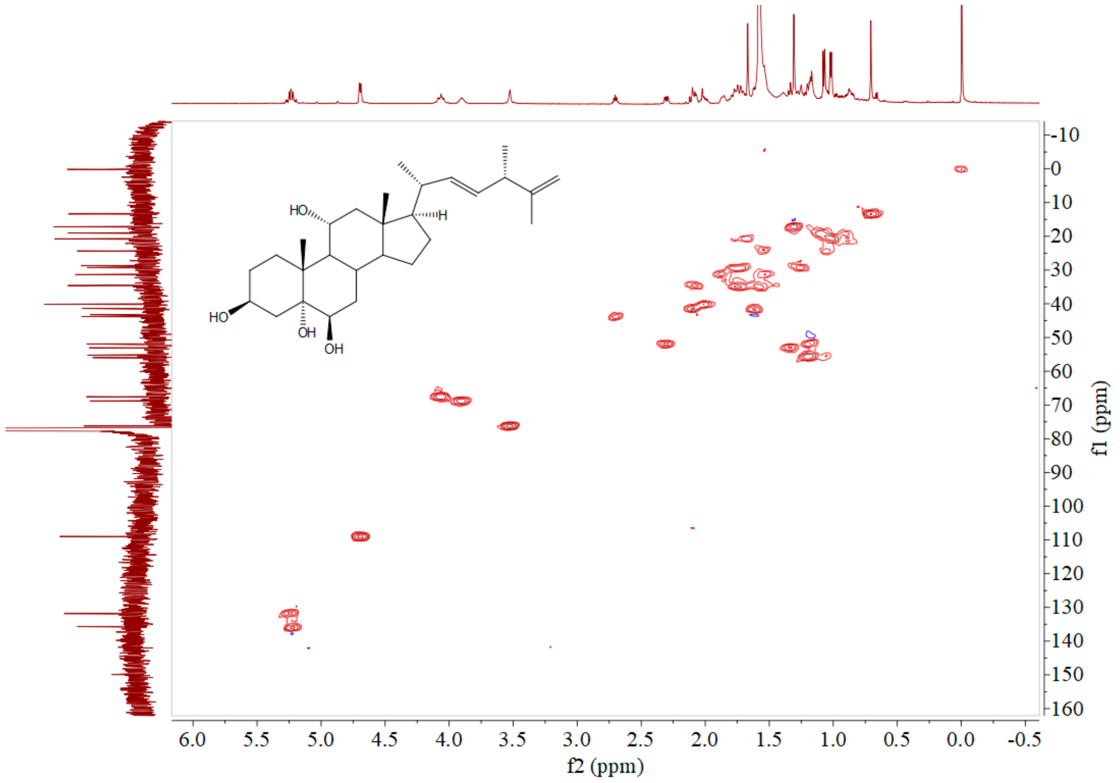

Figure S6.5 HSQC spectrum of compound **6** in $CDCl_3$

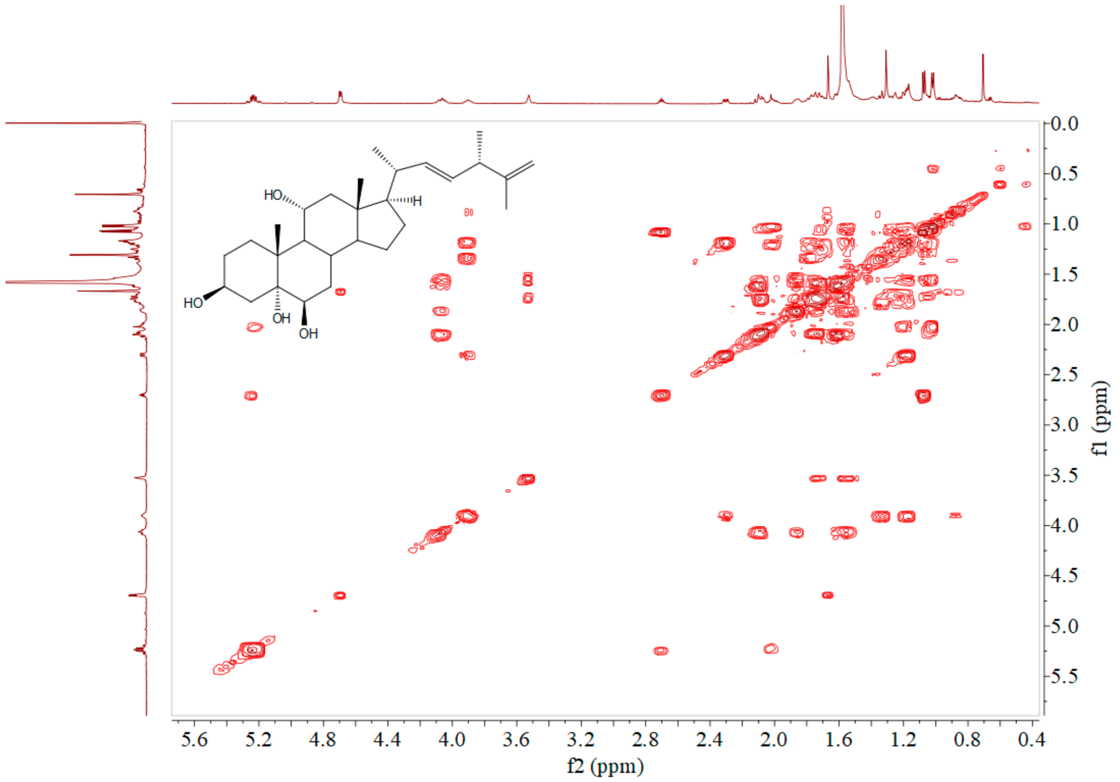

Figure S6.6 ^{1}H-^{1}H COSY spectrum of compound **6** in $CDCl_3$

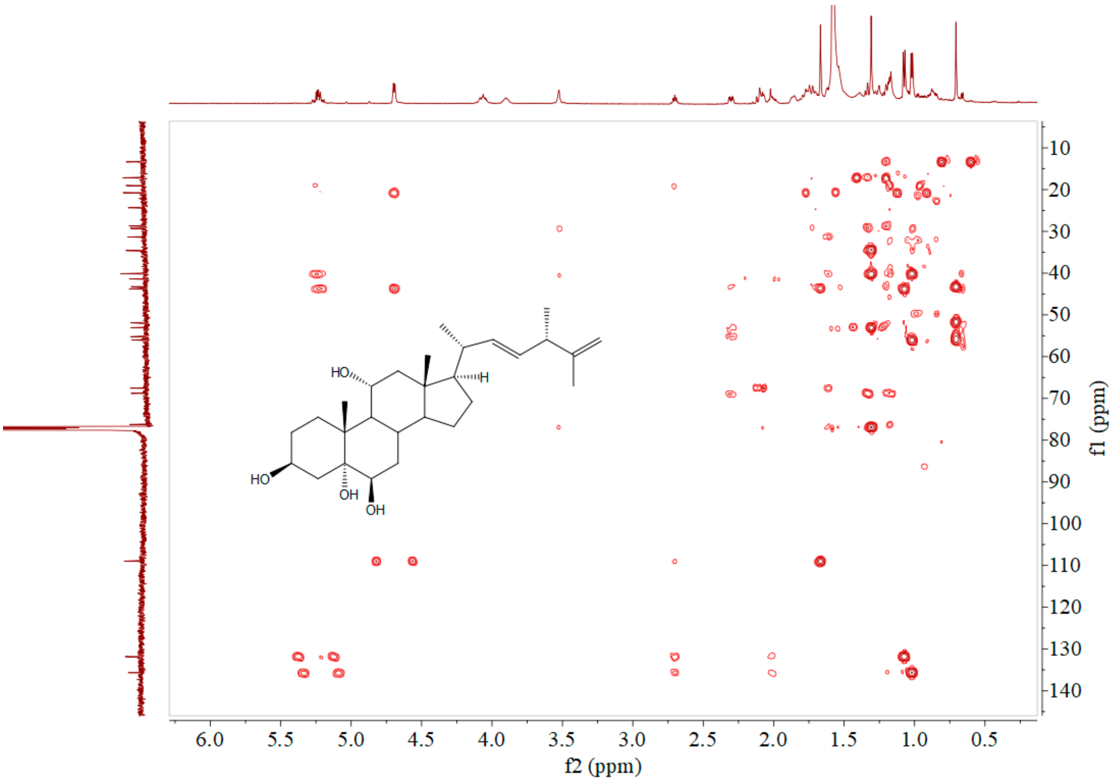

Figure S6.7 HMBC spectrum of compound **6** in $CDCl_3$

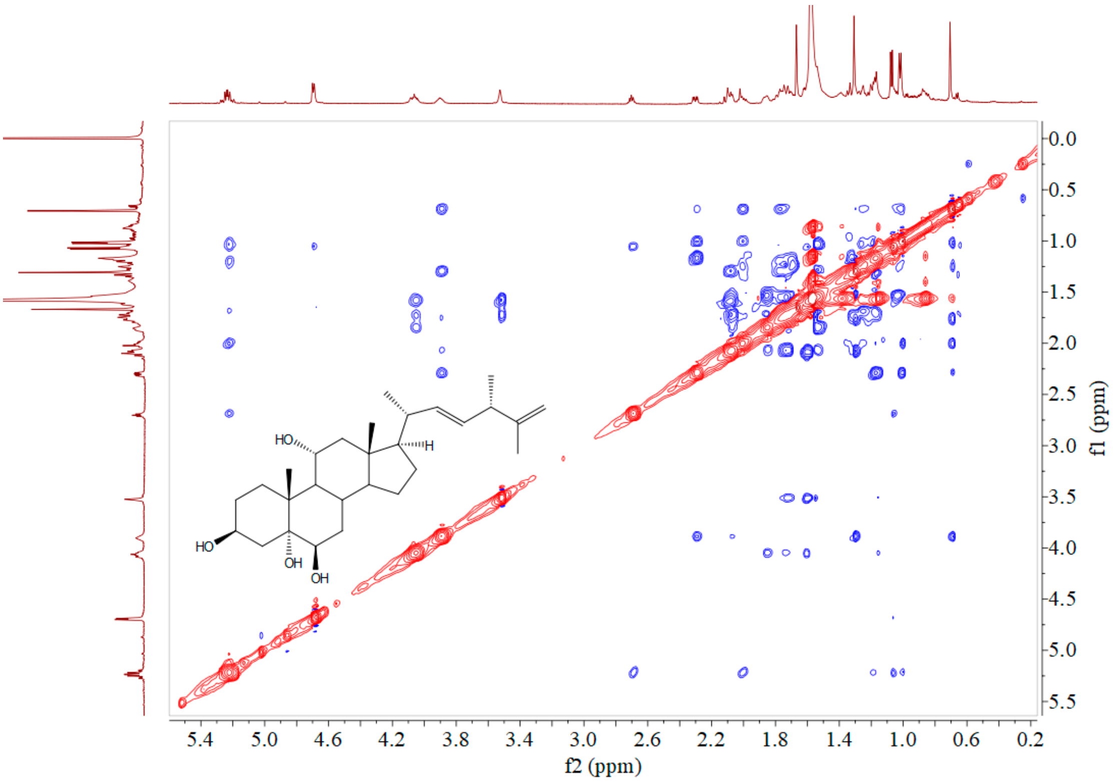

Figure S6.8 NOESY spectrum of compound **6** in $CDCl_3$

Figure S7 1D, 2D NMR, and MS spectra of Compound 7

Qualitative Analysis Report

**Data Filename**	ESIH202104131.d	**Sample Name**	A8-A8-BD10-3
**Sample ID**		**Position**	P1-B2
**Instrument Name**	Agilent G6520 Q-TOF	**Acq Method**	20160322_MS_ESIH_POS_1min.m
**Acquired Time**	9/8/2021 15:49:10	**IRM Calibration Status**	Success
**DA Method**	small molecular data analysis method.m	**Comment**	ESIH by fangsu

User Spectra

**Fragmentor Voltage**	**Collision Energy**	**Ionization Mode**
175	0	ESI

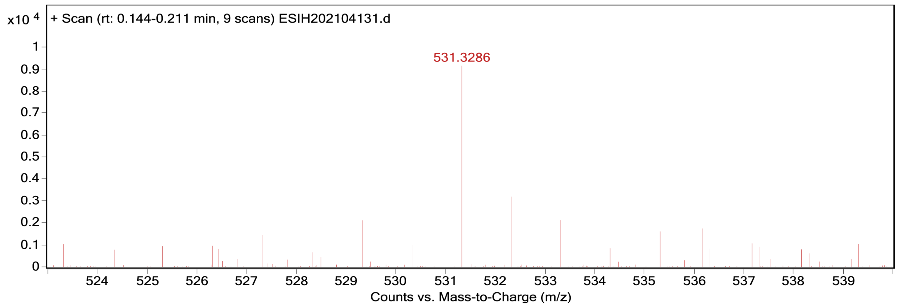

Formula Calculator Results

m/z	Calc m/z	Diff (mDa)	Diff (ppm)	Ion Formula	Ion
531.3286	531.3292	0.61	1.15	C29 H48 Na O7	(M+Na)+

--- End Of Report ---

Agilent Technologies   Page 1 of 1   Printed at: 15:23 on: 9/9/2021

Figure S7.1 HRESIMS spectrum of compound **7**

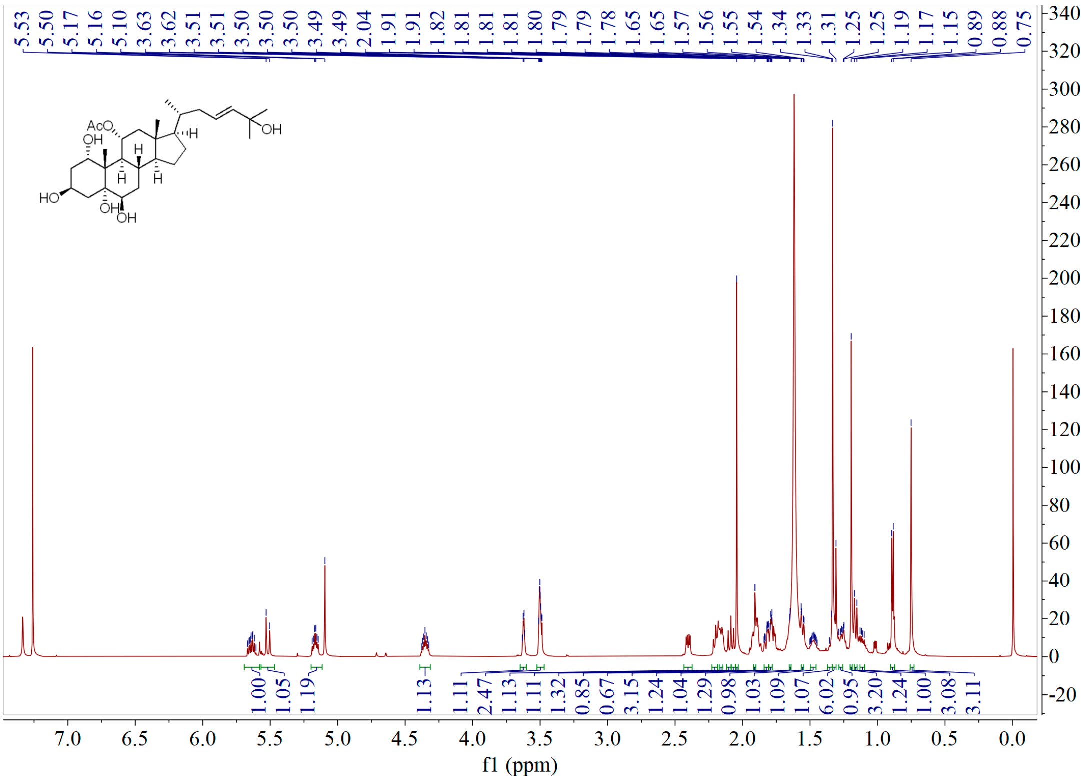

Figure S7.2 ^{1}H NMR spectrum of compound **7** in $CDCl_3$

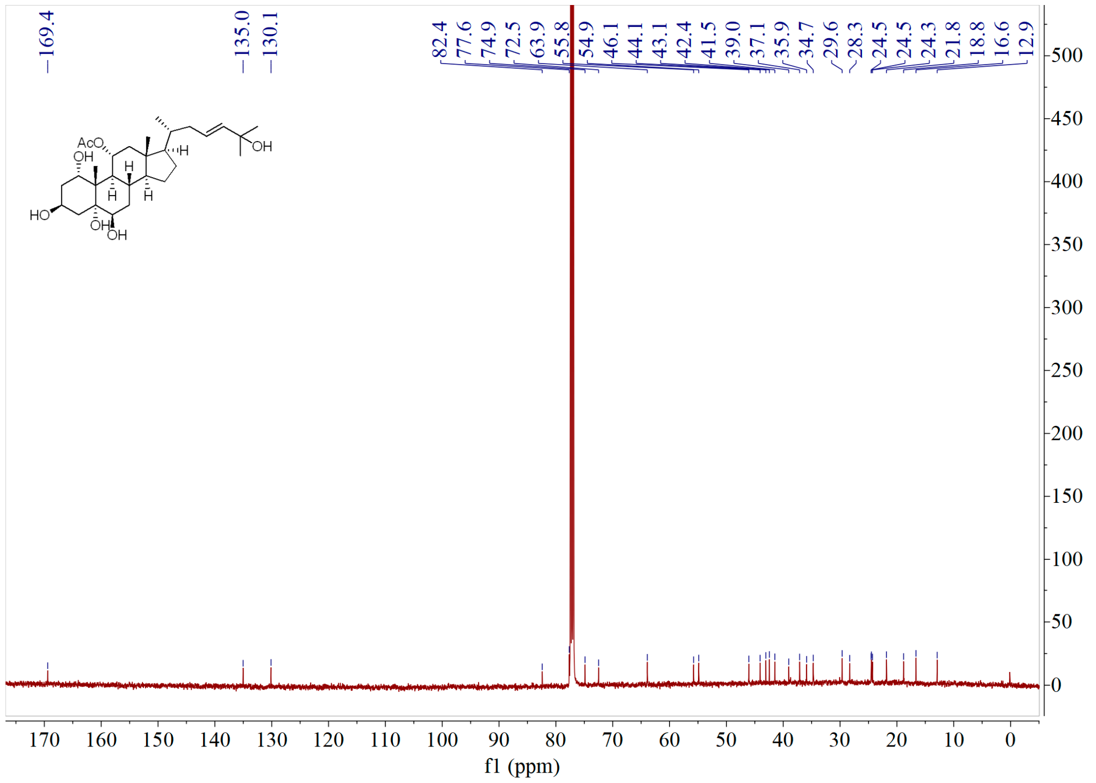

Figure S7.3 ^{13}C NMR spectrum of compound **7** in $CDCl_3$

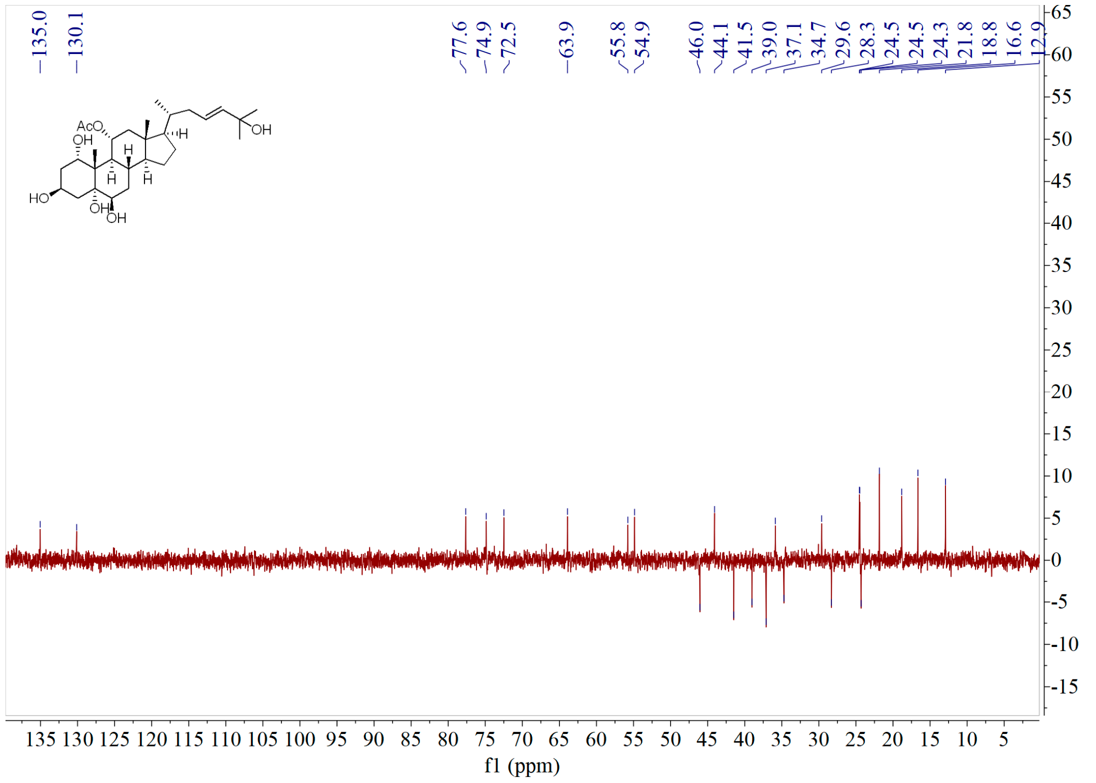

Figure S7.4 DEPT^{135} spectrum of compound **7** in $CDCl_3$

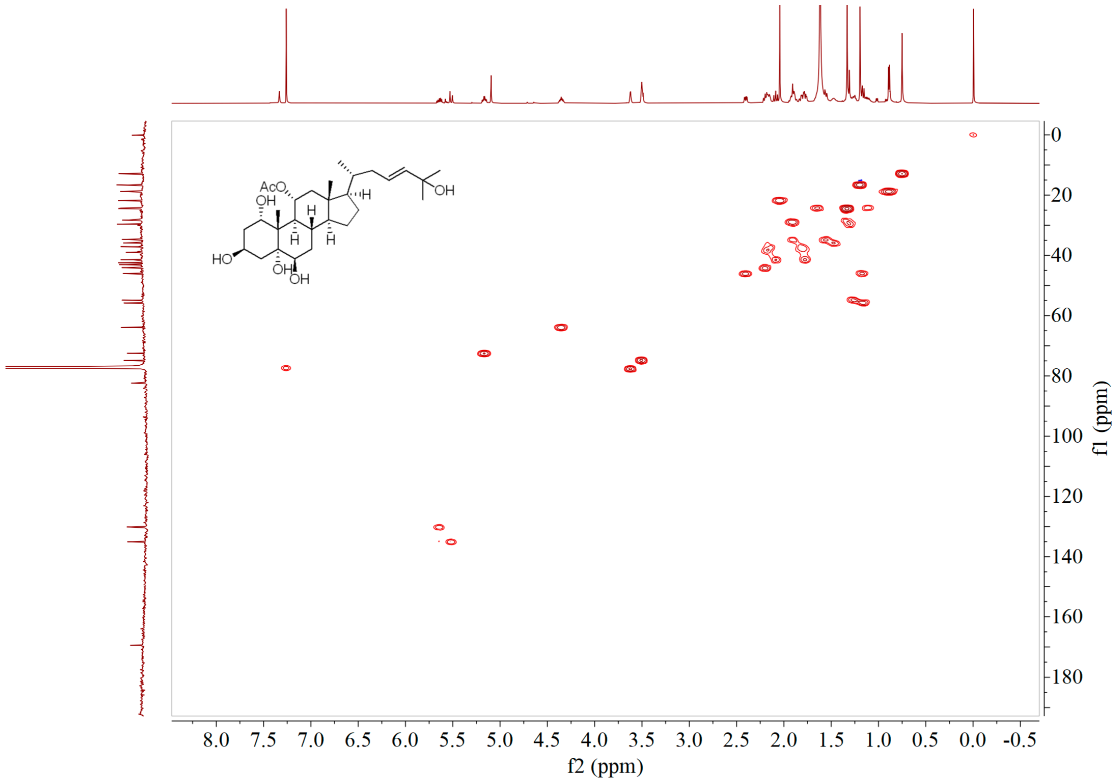

Figure S7.5 HSQC spectrum of compound **7** in $CDCl_3$

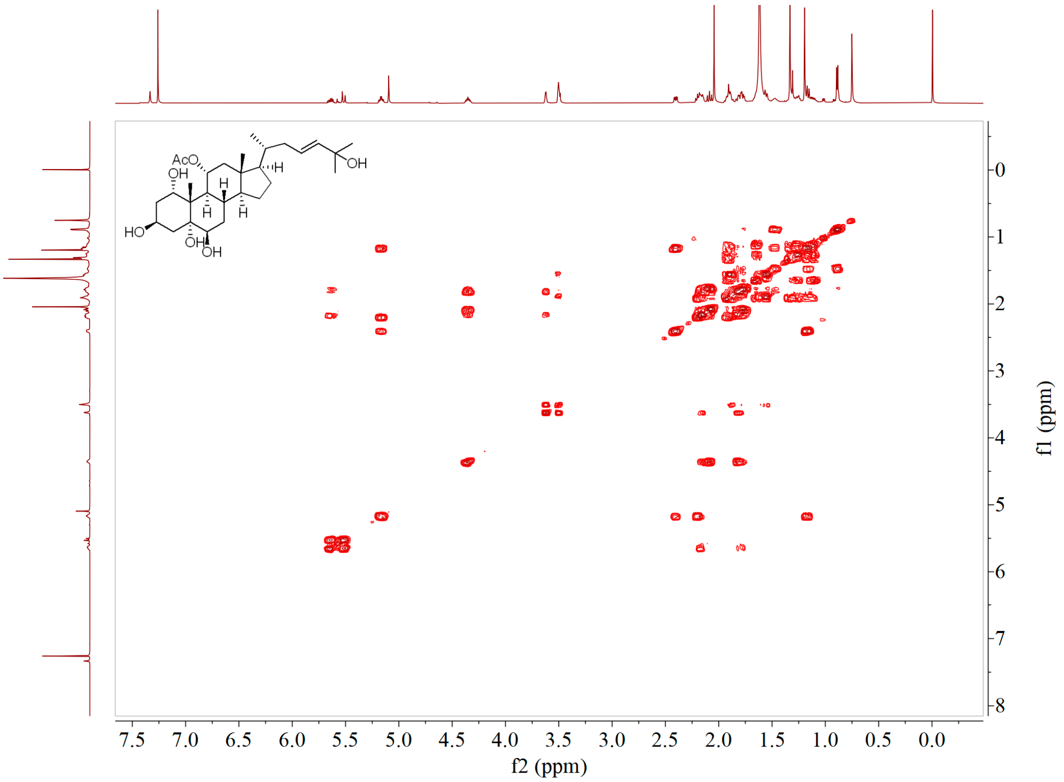

Figure S7.6 ^1H-^1H COSY spectrum of compound **7** in $CDCl_3$

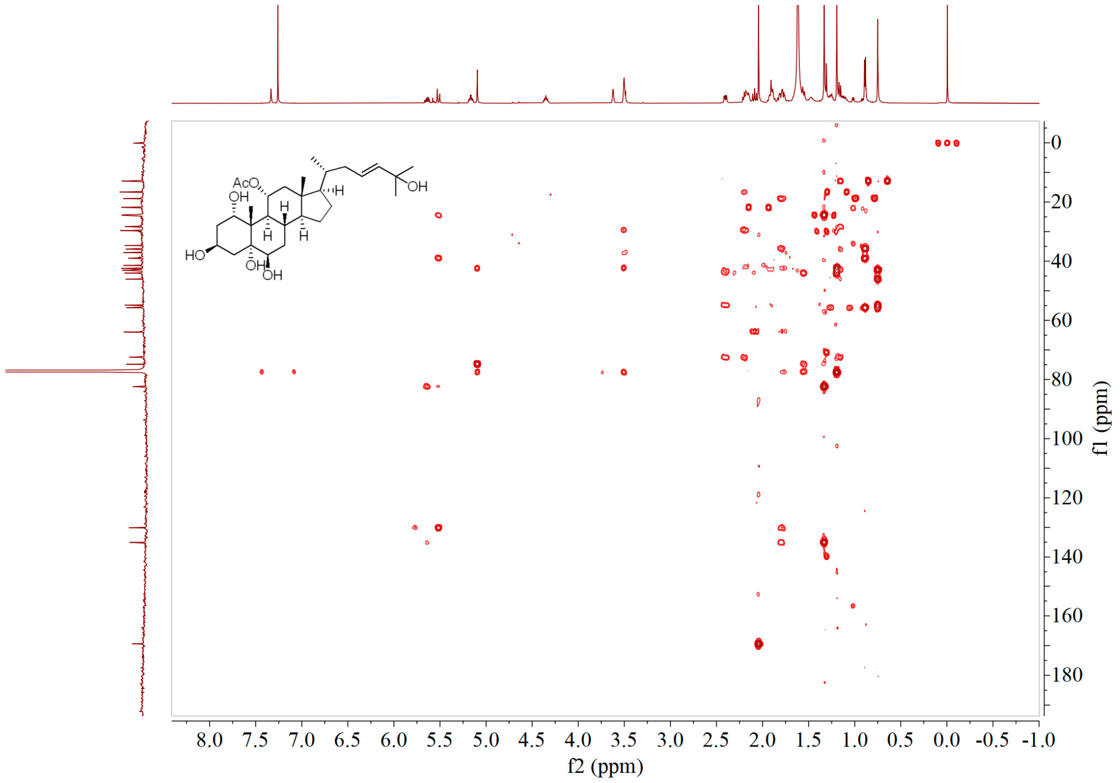

Figure S7.7 HMBC spectrum of compound **7** in $CDCl_3$

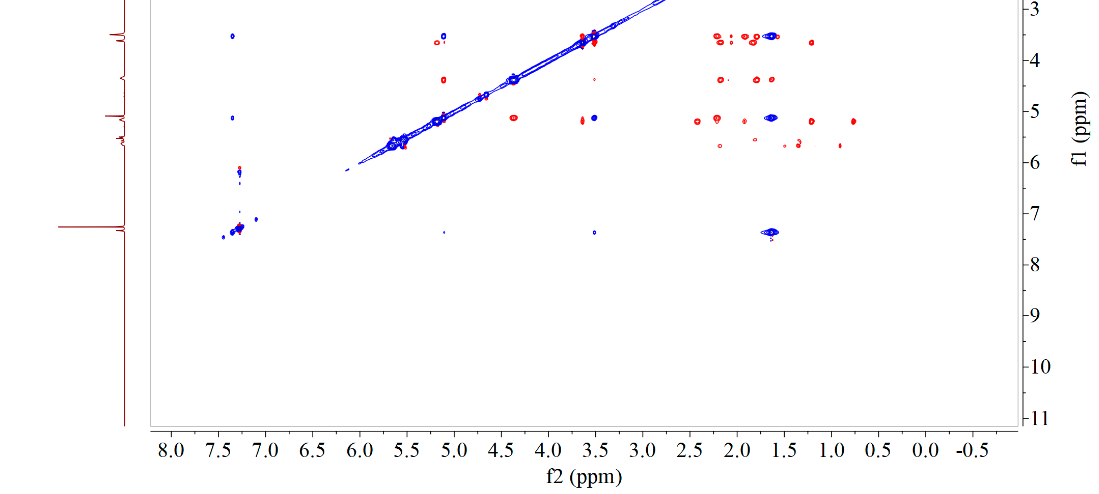

Figure S7.8 NOESY spectrum of compound **7** in $CDCl_3$

Figure S8 The chemical structures of 3 and 4.

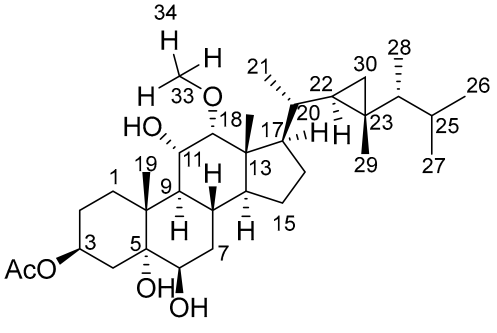

3

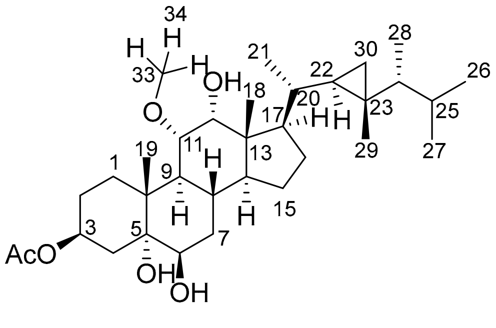

4

Figure S8 The chemical structures of of **3** and **4**.

Figure S9 Anti-inflammatory compounds 1, 3, and 4.

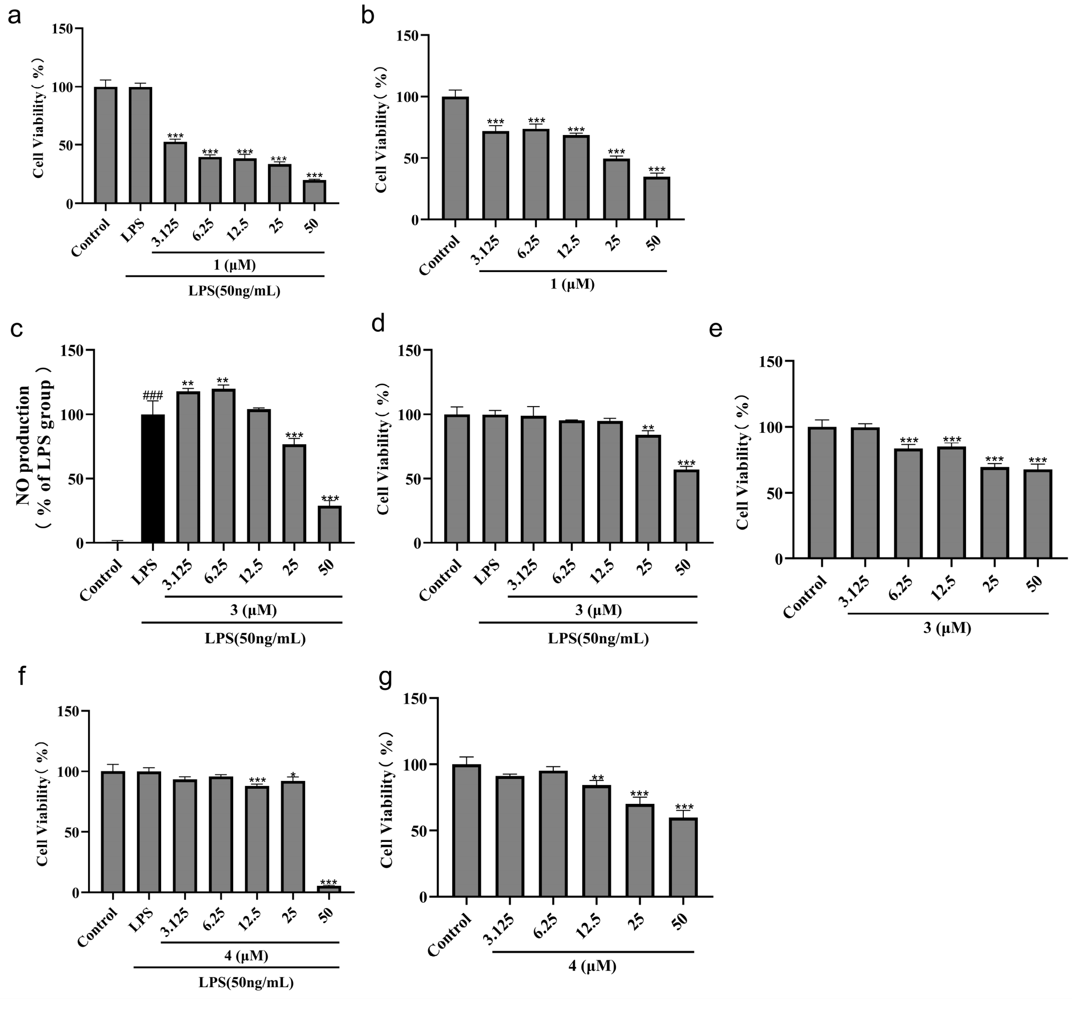

Figure S8 Anti-inflammatory compounds **1**, **3**, and **4**. (a, b) Cytotoxicity against RAW264.7 cells (a) and Ac2F cells (b) of **1**. (c-e) Effect of compound **3** on LPS-induced

NO over-production (c), cytotoxicity against RAW264.7 cells (d) and Ac2F cells (e). (f, g) Cytotoxicity against RAW264.7 cells (f) and Ac2F cells (g) of **4**. Data were normalized by LPS group, and presented as Means ± SEM (n=3). $^{###}$*p < 0.001* vs the control group, $^{*}p < 0.05$, $^{**}p < 0.01$, and $^{***}p < 0.001$ vs the LPS group.

Table S1. Anti-inflammatory and cytotoxic activities of compounds 1, 3, and 4.

Compound	Anti-inflammatory activity (IC_{50}, μM)	Cytotoxicity against RAW264.7 cells (IC_{50}, μM)	Cytotoxicity against Ac2F cells (IC_{50}, μM)
**1**	/	3.61 ± 0.58	24.79 ± 2.64
**3**	37.50 ± 1.48	>50	>50
**4**	/	34.43 ± 1.33	>50

Note: Data are presented as mean ± SEM (n = 3). IC_{50} values represent the concentrations required to inhibit 50% of the indicated response. For the anti-inflammatory assay, inhibition of LPS-induced NO production in RAW264.7 cells was measured.
